# Exploring the Diversity of Mechanisms Associated With Plant Tolerance to Virus Infection

**DOI:** 10.3389/fpls.2018.01575

**Published:** 2018-11-02

**Authors:** Dinesh Babu Paudel, Hélène Sanfaçon

**Affiliations:** ^1^Department of Botany, The University of British Columbia, Vancouver, BC, Canada; ^2^Summerland Research and Development Centre, Agriculture and Agri-Food Canada, Summerland, BC, Canada

**Keywords:** plant–virus interactions, antiviral defenses, disease tolerance, RNA silencing, salicylic acid

## Abstract

Tolerance is defined as an interaction in which viruses accumulate to some degree without causing significant loss of vigor or fitness to their hosts. Tolerance can be described as a stable equilibrium between the virus and its host, an interaction in which each partner not only accommodate trade-offs for survival but also receive some benefits (e.g., protection of the plant against super-infection by virulent viruses; virus invasion of meristem tissues allowing vertical transmission). This equilibrium, which would be associated with little selective pressure for the emergence of severe viral strains, is common in wild ecosystems and has important implications for the management of viral diseases in the field. Plant viruses are obligatory intracellular parasites that divert the host cellular machinery to complete their infection cycle. Highjacking/modification of plant factors can affect plant vigor and fitness. In addition, the toxic effects of viral proteins and the deployment of plant defense responses contribute to the induction of symptoms ranging in severity from tissue discoloration to malformation or tissue necrosis. The impact of viral infection is also influenced by the virulence of the specific virus strain (or strains for mixed infections), the host genotype and environmental conditions. Although plant resistance mechanisms that restrict virus accumulation or movement have received much attention, molecular mechanisms associated with tolerance are less well-understood. We review the experimental evidence that supports the concept that tolerance can be achieved by reaching the proper balance between plant defense responses and virus counter-defenses. We also discuss plant translation repression mechanisms, plant protein degradation or modification pathways and viral self-attenuation strategies that regulate the accumulation or activity of viral proteins to mitigate their impact on the host. Finally, we discuss current progress and future opportunities toward the application of various tolerance mechanisms in the field.

## Introduction

**FIGURE 1:**
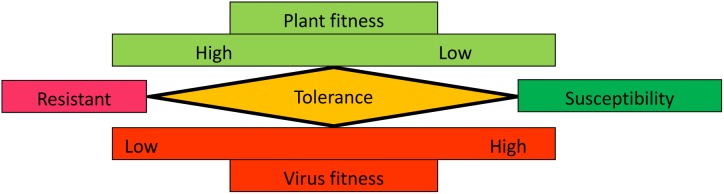
Graphical representation of plant and virus fitness in resistant, tolerant, or susceptible interactions. See text for details.

Tolerance to biotic stresses caused by pathogens, including viruses, is well-documented in plants (Rausher, 2001; Pagan and Garcia-Arenal, 2018). Tolerance has been defined as a mitigation of the impact of virus infection irrespective of the pathogen load (Cooper and Jones, 1983). Although a significant virus load is sustained, the plant growth, yield or reproduction attributes are only minimally affected and visible symptoms are either absent or mild. Tolerance can be explained as reaching equilibrium to allow acceptable compromises in host and virus fitness for long-term co-existence (Figure 1). Because viruses are intracellular obligate parasites, they require host resources to complete their infection cycle (Culver and Padmanabhan, 2007; Nagy and Pogany, 2012; Wang, 2015). Therefore, high virus fitness is at the expense of the host in symptomatic susceptible interactions. In resistant interactions, the plant fitness is preserved by preventing virus accumulation or systemic movement. In tolerant interactions, virus fitness is reduced by preventing over-accumulation of viral RNAs or by minimizing the concentration or activity of viral proteins that play a role in virulence. In turn, this limits the damage to the host. Because of their absolute dependence on their host, maintaining host fitness is also beneficial to viruses.

Plant viruses should not only be viewed as pathogens. In fact, experimental evidence documenting the beneficial impact of accommodating long-term virus infection is accumulating, especially in natural environments (Roossinck, 2011; Roossinck and Bazan, 2017). Indeed, virus infection can improve the plant resilience in sub-optimal environmental conditions, for example tolerance to drought. Virus-induced drought tolerance is associated with global reprogramming of plant gene expression, changes in hormone signaling and increased accumulation of metabolites and antioxidants (Xu et al., 2008; Westwood et al., 2013; Aguilar et al., 2017; Dastogeer et al., 2018). Interestingly, recent studies suggested that the benefits of increased drought resistance can be offset by increased virus virulence (Aguilar et al., 2017; Berges et al., 2018). Maintaining persistent virus infection can also improve the plant resistance to biotic stress including non-vector herbivory insects, other viruses, or unrelated pathogens (van Molken et al., 2012; Shapiro et al., 2013; Mascia and Gallitelli, 2016; Syller and Grupa, 2016). Thus, tolerance to virus infection does not only mitigate the impact on the host as shown in Figure 1, but under additional abiotic or biotic stress, it can actually enhance the host fitness. In agricultural settings, tolerance is often effective against a larger spectrum of isolates compared to resistance (Korbecka-Glinka et al., 2017). Because viruses are allowed to persist, the selection pressure for emergence of virulent strains is also reduced in tolerant cultivars compared to resistant cultivars (Rausher, 2001; Pagan and Garcia-Arenal, 2018). Thus, tolerance can be considered as an evolutionary stable defense response.

While many plant antiviral resistance genes (R genes) have been characterized (de Ronde et al., 2014; Miyashita and Takahashi, 2015; Sanfacon, 2015; Hashimoto et al., 2016), the genetic basis of tolerance is much less well-understood. However, tolerance and resistance are not necessarily mutually exclusive in the field and mechanisms that govern both outcomes can overlap significantly (Pagan and Garcia-Arenal, 2018). In fact, many defense responses genes that are activated by dominant R genes are also induced in tolerant interactions (Bengyella et al., 2015). As will be detailed below, tolerance is often explained by the balance between plant antiviral mechanisms and viral counter-defense responses.

A recent review focused on plant–pathogen co-evolution in tolerant interactions (Pagan and Garcia-Arenal, 2018). In this review, we explore the molecular mechanisms that are associated with plant tolerance to virus infection. This review is not meant as an encyclopedic list of all known aspects of plant–virus interactions, rather we have selected examples that illustrate the variety of mechanisms that help attain long-term tolerance to virus infection. We also discuss current knowledge gaps as well as progress and future opportunities toward applications in the field.

## Plant Antiviral Defense and Virus Counter-Defenses

The majority of plant viruses are considered generalists as they can infect a large variety of plant hosts (García-Arenal and Fraile, 2013). However, this does not mean that plants are passive in their interactions with viral pathogens. Indeed, although plants do not possess an equivalent to the animal adaptive immune system, they deploy a number of protein- and RNA-mediated defense mechanisms against viruses (Zvereva and Pooggin, 2012; Mandadi and Scholthof, 2013; Moon and Park, 2016; Gouveia et al., 2017; Nicaise, 2017; Carr et al., 2018). In turn, viruses have developed sophisticated counter-defenses to allow systemic infection of plants. The balance between plant defense responses and viral counter-defenses is finely tuned, often allowing the virus to persist without causing too much damage to its host.

### Antiviral RNA Silencing

RNA silencing is often considered the most important basal adaptive plant antiviral defense response (Moon and Park, 2016). RNA silencing is a ubiquitous gene regulation mechanism, which is based on the generation of small RNAs that guide the silencing machinery to complementary nucleic acids for transcriptional gene silencing (TGS) or post-transcriptional gene silencing (PTGS) (Martinez de Alba et al., 2013). TGS results in the methylation and transcription repression of target DNAs, while PTGS operates by slicing target RNAs or repressing their translation. Plant DICER-like (DCL) proteins recognize double-stranded RNA (dsRNA) structures and process them into 21–25 nucleotides small RNA duplexes (Borges and Martienssen, 2015). One of the small RNA strands, the guiding strand, is loaded into ARGONAUTE (AGO) protein-containing RNA-induced silencing complexes (RISC) or RNA-induced transcriptional silencing complexes (RITSs) and directs these complexes to target nucleic acids in a sequence-specific manner for PTGS or TGS, respectively. In the context of antiviral RNA silencing, DCL enzymes recognize dsRNA structures present in replication intermediates produced during the replication of RNA viruses, in hairpin structures of viral RNAs, or in aberrant viral dsRNAs amplified by plant RNA-dependent RNA polymerases to produce viral-derived small interfering RNAs (vsiRNA), which are incorporated in RISC or RITS complexes (Raja et al., 2010; Martinez de Alba et al., 2013; Csorba et al., 2015; Ghoshal and Sanfacon, 2015; Zhang C. et al., 2015; Ramesh et al., 2017). Plant microRNAs (miRNAs) are produced after processing of folded endogenous plant mRNAs derived from miRNA genes by DCL enzymes and are also highly relevant to plant–virus interactions (Martinez de Alba et al., 2013; Cui et al., 2017). As will be described below, specific miRNAs regulate the expression of genes coding for RNA silencing enzymes or other defense proteins.

Most viruses encode a viral suppressor of silencing (VSR) to counteract the plant antiviral RNA silencing. Characterized VSRs show tremendous diversity in their protein sequence and mode of actions (Csorba et al., 2015). VSRs can block RNA silencing by inhibiting the initiation/spread of RNA silencing (e.g., by binding small RNAs and sequestering them away from the silencing complexes), by affecting the assembly/stability/function of silencing complexes (e.g., by destabilizing or inhibiting AGO proteins) or by redirecting silencing complexes in the regulation of host defense genes (e.g., by inducing the transcription of endogenous miRNAs that down-regulate key plant silencing factor genes) (Csorba et al., 2015; Wieczorek and Obrepalska-Steplowska, 2015). VSRs can specifically disrupt PTGS or TGS or can simultaneously affect both. Interestingly, some VSRs function by interacting with endogenous plant suppressors of silencing and/or by activating their transcription (Trinks et al., 2005; Endres et al., 2010; Yong Chung et al., 2014). Finally, it should be noted that some viruses encode more than one VSR (Lu et al., 2004) and that some VSRs can target multiple steps of RNA silencing (Csorba et al., 2015).

### Salicylic Acid-Mediated Defense Responses

Salicylic acid (SA) is a key signal molecule in plants that mediates defense responses associated with basal innate immunity and with inducible immunity directed by antiviral dominant R genes (Mandadi and Scholthof, 2013; Gouveia et al., 2017; Carr et al., 2018). Basal innate immunity associated with bacterial and fungal infection depends on surface-associated receptors that recognize conserved microbe/pathogen-associated molecular patterns (M/PAMPs) and induce a cascade of events leading to PAMP- triggered immunity (PTI) (Jones and Dangl, 2006). In the case of virus infection, the presence of intracellular dsRNAs has been shown to trigger the PTI response in plants independently of the RNA silencing pathway (Niehl et al., 2016). PTI is accompanied with SA accumulation, and triggers a cascade of events, including an oxidative burst, activation of mitogen-activated kinases and induced expression of defense genes (e.g., pathogenesis-related or PR proteins) (Bigeard et al., 2015).

The second line of SA-mediated defense responses is often referred to as the effector-triggered defense (ETI). ETI requires the recognition of pathogen avirulent proteins, also termed effectors, by plant intracellular receptors, which are encoded by dominant R genes (Jones and Dangl, 2006). Most known antiviral dominant R genes encode proteins with nucleotide-binding leucine-rich repeats (NB-LRR) that share similarities with R genes directed at fungal and bacterial pathogens (Moon and Park, 2016; Gouveia et al., 2017). The ETI defense response is similar to PTI in its nature, but is more acute. ETI is generally associated with a local hypersensitive reaction (HR), which causes rapid cell death and the formation of visible necrotic lesions on inoculated leaves, and with the subsequent establishment of systemic acquired resistance (Moon and Park, 2016; Gouveia et al., 2017).

Several plant viruses have been shown to suppress the oxidative burst and the expression of defense genes associated with PTI or ETI (Hussain et al., 2007; Mubin et al., 2010; Zvereva et al., 2016; Nicaise and Candresse, 2017). A replicase protein of tobacco mosaic virus promotes the degradation of ATF2, a plant NAC transcription factor, which regulates the expression of PTI-responsive genes (Wang X. et al., 2009). Similarly, interaction between the turnip crinkle virus coat protein (CP) and TIP, another NAC transcription factor was correlated with the inhibition of innate immune defense responses (Donze et al., 2014). Finally, the P6 protein from cauliflower mosaic virus (CaMV) suppresses SA-signaling in part by modulating the expression and sub-cellular localization of NPR1 (NON-EXPRESSOR OF PATHOGENESIS-RELATED1), a transcriptional activator of downstream SA-responsive genes (Love et al., 2012).

### Dominant or Recessive Antiviral Resistance Genes That Do Not Depend on SA Signaling

Some characterized dominant R genes do not encode proteins with signature NB-LRR sequences and do not induce ETI-like defense responses (Gouveia et al., 2017). These R genes limit virus infection using different mechanisms. For example, a protein encoded by the tomato *Tm-1* gene binds to the tomato mosaic virus replication proteins and inhibits viral RNA replication (Ishibashi and Ishikawa, 2014). Finally, there are many characterized plant recessive resistance genes that correspond to mutations of plant factors that are essential to the virus infection cycle, most often translation factors, such as eIF4E or eIF4G (Truniger and Aranda, 2009; Sanfacon, 2015; Hashimoto et al., 2016).

### Role of Plant Hormones in Antiviral Defenses and Cross-Talks Between Defense Mechanisms

In addition to RNA silencing and SA-mediated defenses, signaling pathways controlled by various plant hormones influence plant antiviral responses (reviewed in Robert-Seilaniantz et al., 2011; Mandadi and Scholthof, 2013; Alazem and Lin, 2015; Carr et al., 2018). Jasmonic acid (JA) and ethylene (Et) are normally associated with defense mechanisms that operate against necrotrophic pathogens (JA and Et) and insect pests (JA) and have antagonistic effects on SA signaling and associated defense responses. Abscisic acid (ABA) regulates plant development and modulates the response to environmental stresses. ABA also has antagonistic effects on the SA- and JA/Et-pathways. Multiple levels of cross-talk among the SA-, JA-, ABA-signaling pathways and RNA silencing highlight complex regulatory mechanisms of host defense responses that are manipulated by viruses to their advantage. For example, some VSRs interfere not only with antiviral RNA silencing but also with the SA-, JA- or Et-pathways, in some cases down-regulating plant defense responses to promote their transmission by insect vectors (Ji and Ding, 2001; Geri et al., 2004; Lozano-Duran et al., 2011; Love et al., 2012; Westwood et al., 2014; Zvereva et al., 2016; Wu et al., 2017; Poque et al., 2018). SA was also recently shown to regulate cross-talks between gibberellin synthesis/signaling (involved in plant development) and the induction of miRNAs targeting plant defense genes (Kriznik et al., 2017). Finally, primary plant metabolism pathways (synthesis of carbohydrates, lipids, or amino acids) have been shown to impact plant defense responses (Bolton, 2009; Rojas et al., 2014). For example, sugars are both essential energy resources for the activation of defense responses and regulators of these responses (Bolouri Moghaddam and Van den Ende, 2012).

## Symptom Determinants in Plant–Virus Interactions

### Fitness Costs of Activating the Plant ETI or PTI Defense Responses

Expression of defense genes during ETI or PTI is associated with fitness costs. As mentioned above, mounting the defense response requires energy resources, which are diverted at the expense of plant growth and development. Indeed, constitutive overexpression of R genes or other defense genes often causes pleiotropic effects on plant development (Heil and Baldwin, 2002; Tian et al., 2003; Yang and Hua, 2004; Yi and Richards, 2007). Induction of defense hormones can also result in reduced plant growth (Huot et al., 2014; Havko et al., 2016; Guo et al., 2018). Thus, the activation of SA-dependent defense responses is likely one of the factors contributing to the dwarfing phenotypes observed in many plant–virus interactions.

Plants down-regulate the expression of R genes or associated defense genes in the absence of pathogen pressure using either dedicated repressor genes or miRNA-mediated RNA silencing. For example, the *Arabidopsis thaliana BONZAI1* (*BON1*) gene down-regulates the expression of the R-like gene *SNC1* (Yang and Hua, 2004). Plant miRNAs have been identified that target characterized R genes or R-like genes with signature NB-LRR sequences (He et al., 2008; Zhai et al., 2011; Li et al., 2012; Shivaprasad et al., 2012; Deng et al., 2018). These miRNAs often target conserved regions of R or R-like genes resulting in the production of secondary siRNAs, which in turn down-regulate a larger number of related genes based on sequence similarities (Zhai et al., 2011; Li et al., 2012; Shivaprasad et al., 2012; Boccara et al., 2014). Following virus infection, the miRNA-mediated repression of R and R-like genes is released and the plant defense is upregulated (Shivaprasad et al., 2012). This may be an indirect consequence of the inhibition of plant RNA silencing by VSRs. Indeed, elevated expression of the R-like gene *SNC1* is observed in plants expressing VSRs (Yi and Richards, 2007). Similarly, tobacco plants expressing the potyvirus HC-Pro VSR display enhanced resistance to various pathogens, including several viruses (Pruss et al., 2004; Jovel et al., 2011). As a counter-defense, some plant viruses regulate the expression of specific miRNAs that target R or R-like genes (e.g., miR1885 induced by turnip mosaic virus) (He et al., 2008), or other defense genes (miR164a that targets NAC transcription factors implicated in regulating cell death) (Bazzini et al., 2009).

Necrotic responses associated with HR are generally thought to play a role in restricting virus movement. However, HR is not always efficient at restricting viruses and cells outside of the cell death zone of local necrotic lesions can harbor infectious virus (Lukan et al., 2018). In some pathosystems, induction of HR is either weak or delayed and does not prevent the systemic spread of viruses. Instead, this can result in runaway HR leading to systemic lethal necrosis (Moffett, 2009; Pallas and Garcia, 2011; Mandadi and Scholthof, 2013; Künstler et al., 2016).

### Impact of Viral Infection on Plant Organelles

In susceptible plants, virus infection can cause profound reorganization of host cells, by altering the structure and integrity of intracellular membranes and organelles (Laliberte and Sanfacon, 2010). A common symptom of virus infection is chlorosis, often expressed as yellow mosaic symptoms on the leaves. Chlorotic symptoms have been correlated with virus-induced changes in the number or size of chloroplasts, or with structural alterations: invaginations of chloroplast membranes, formation of tubular stromules, changes in the number or appearance of grana or starch grains (Li et al., 2016; Zhao et al., 2016; Bhattacharyya and Chakraborty, 2017). In addition, biotic stress including viral infection has been reported to cause global repression of plant photosynthetic genes (Bilgin et al., 2010). The chloroplast is a key player in the deployment of plant defense responses with SA, JA, and reactive oxygen species being produced in the chloroplast (Dempsey et al., 2011; Kangasjarvi et al., 2012; Li et al., 2016; Zhao et al., 2016; Bhattacharyya and Chakraborty, 2017). It was recently shown that ETI-dependent activation of MPK3/MPK6 (mitogen-activated kinases) inhibits photosynthesis which in turn leads to the accumulation of reactive oxygen species required for the HR (Su et al., 2018). Specific interactions between viral and chloroplast proteins can also interfere with the normal functioning of the chloroplast (Zhao et al., 2016).

Replication of RNA viruses requires association with and extensive modification of intracellular membranes derived most often from the endoplasmic reticulum (ER), but also from chloroplasts, peroxisomes or vacuoles, depending on the specific plant–virus interaction (Laliberte and Sanfacon, 2010; Jin et al., 2018). Cell-to-cell movement of some viruses also require modification of ER membranes. The ER is an important organelle that orchestrates post-translational modifications and folding of cellular proteins destined to the secretory system. Alterations of the ER structure caused by virus infection and the vigorous ER-associated synthesis of viral proteins can cause severe ER stress, which if not mitigated, can lead to programmed cell death (Zhang and Wang, 2012; Verchot, 2016a). Most often, viral integral membrane proteins are responsible for the ER modifications. In some cases, these viral proteins act as viroporins, creating aqueous pores in the membranes and affecting their integrity (Nieva et al., 2012; Sanfacon, 2013). In addition, viral movement proteins (MPs) interact with and modify the plasmodesmata that connect plant cells to promote virus cell-to-cell movement, a process which can disrupt the natural movement of nutrients and signal molecules between cells (Harries and Ding, 2011). Alterations of the actin and tubulin intracellular transport networks are also common consequences of plant virus infection (Niehl et al., 2013; Pitzalis and Heinlein, 2017).

### Toxic Effects of Viral Proteins

In addition to the gross alterations in sub-cellular structures described above, a large network of interactions between plant and virus proteins has been characterized (Wang, 2015; Nagy, 2016). In fact, hub viral proteins may interact with a large number of host proteins. For example, the tombusvirus p33 replication protein has more than 100 known plant protein interaction partners (Nagy, 2016). Although, it is beyond the scope of this review to describe each known protein-protein interaction, it is important to note that many of these interactions affect the host physiology profoundly, which can lead to visual symptoms and/or impact the host general fitness (reviewed in Culver and Padmanabhan, 2007; Mandadi and Scholthof, 2013).

Although many viral proteins contribute to virulence, VSRs are often virulence factors and symptom determinants. VSRs were first discovered in the context of synergistic interactions between two plant viruses. The potyvirus HC-Pro protein was shown to assist a potexvirus with counter-defense responses to the plant antiviral RNA silencing, resulting in increased symptom severity (Anandalakshmi et al., 1998). The virulence properties of VSRs may be partly due to the increased virus accumulation that follows the inhibition of the plant antiviral RNA silencing. However, symptom severity is not always correlated with the level of genomic viral RNA accumulation (Pagan et al., 2007). For example, a chimeric potato virus X expressing the potyvirus HC-Pro VSR accumulates to lower levels than the native virus in infected plants but causes more severe symptoms (Aguilar et al., 2015). Several VSRs are also recognized as elicitors of dominant R genes and trigger necrotic defense responses (Li et al., 1999; Wang et al., 2015). Because RNA silencing is a ubiquitous gene regulation mechanism in plants, VSRs may disturb not only antiviral RNA silencing pathways but also other aspects of the plant metabolism and development that are regulated by RNA silencing. As mentioned above, VSRs can impact the expression, stability or activity of AGO proteins, in particular AGO1 which is required for miRNA-mediated regulation of plant gene expression. Thus, ectopic expression of VSRs in transgenic lines can cause phenotypic changes, similar to symptoms induced during virus infection or to those observed in AGO1-deficient mutants (Zhang et al., 2006; Bortolamiol et al., 2007; Varallyay and Havelda, 2013). Similarly, many VSRs such as the tombusvirus p19 protein have been shown to sequester not only vsiRNAs but also plant siRNAs or miRNAs (Chapman et al., 2004; Wu et al., 2010; Pertermann et al., 2018). Interestingly, recent reports suggest that p19 sequesters vsiRNAs more efficiently than miRNAs and that miRNA binding may only occur early in infection when the concentration of vsiRNAs is still low (Kontra et al., 2016; Pertermann et al., 2018). Thus, the regulation of this VSR activity is fine-tuned during virus infection perhaps to mitigate its impact on the host physiology.

### Viral siRNAs Directed at Plant Genes

Reports on how viruses use vsiRNAs to modulate the expression of plant genes are emerging. *In silico* analysis, and in some cases further functional validation, revealed many plant mRNA targets of vsiRNA in several plant–virus interactions (Qi et al., 2009; Miozzi et al., 2013; Stare et al., 2015; Wang et al., 2016a; Moyo et al., 2017; Xu and Zhou, 2017). Perhaps not surprisingly, several targeted transcripts encode proteins related to host stress responses and signal transduction. For example, vsiRNA of cotton leaf curl Multan virus were shown to target a gene encoding a MYB transcription factor that restrict virus accumulation (Wang et al., 2016a). Targeting of plant genes by vsiRNAs can also cause visual symptoms. Infection of cucumber mosaic virus together with the associated satellite Y RNA causes yellowing of leaves in *Nicotiana tabacum*. This was correlated with the down-regulation of a gene involved in chlorophyll biosynthesis (*ChlI*) which is targeted by small RNAs derived from the satellite RNA (Shimura et al., 2011; Smith et al., 2011). Similarly, downregulation of *Nicotiana benthamiana* eukaryotic translation initiation factor (NbeIF4A) was shown to be associated with the stunting phenotype of *N. benthamiana* plants infected with rice stripe virus (Shi et al., 2015).

## Prevalence of Tolerance in Wild Ecosystems and Impact of Environmental Factors

### Long-Term Mutually Beneficial Co-existence Defines Many Plant–Virus Interactions in Natural Environments

Plant viruses were first discovered because of their impact on economically important crops and as a consequence they have been described as pathogens for many years. However, plant–virus interactions are much more complex in natural environments. Metagenomic studies have revealed that virus infection is common in natural ecological settings with 60–70% of plants infected with one or several viruses (Roossinck et al., 2015). Interestingly, virus-infected plants are normally asymptomatic in wild environments (Roossinck, 2014). In fact, the point has been made that large-scale crop monocultures in agriculture settings and the consequent loss of biodiversity has contributed to the emergence of severe plant virus diseases (Roossinck, 2015; Roossinck and Garcia-Arenal, 2015). In natural settings, generalist viruses would be favored. Accommodating a wider host range usually results in reduced virulence, in part because of selection pressures to evade or counteract multiple defense responses that vary in their intensity from host to host (Miyashita et al., 2016). In the wild, plants and viruses are exposed to long-term ongoing selection pressures from multiple biotic and abiotic stresses (McLeish et al., 2018). Mixed virus infections are common in plants and can result in synergistic or antagonistic interactions (Mascia and Gallitelli, 2016) that also influence virus evolution and adaptation to new hosts (McLeish et al., 2018). The strict requirement of many viruses for vector transmission (most often insects) also drives virus evolution and virulence (Hily et al., 2014; Roossinck, 2015; Blanc and Michalakis, 2016; Hamelin et al., 2017). While viruses may afford to kill or damage their hosts in agricultural settings because of the prevalence of specialized insect vectors adapted to specific crops, extending the lifespan and fitness of the host is a more viable option in natural environments. Finally, it should also be noted that in nature many persistent viruses do not depend on vector transmission (Roossinck, 2014; Roossinck and Bazan, 2017). Rather, they are strictly vertically transmitted through seeds and must ensure successful reproduction of their host. While the prevalence of tolerant and often mutually beneficial interactions in the wild is well-documented, the molecular mechanisms that govern these interactions have not yet been characterized. This will likely become a focus of future research.

### Age-Dependent Tolerance to Virus Infection

Plants exhibit more tolerance to disease as they age. The maintenance of TGS and PTGS can differ in plants that are in vegetative or reproductive stages and some VSRs are not active in older plants at the reproductive stage (Jackel et al., 2015). For example, mature plants show decreased concentration of the small RNAs that regulate the expression of a tobacco R gene directed at tobacco mosaic virus (the *N* gene) (Deng et al., 2018). Furthermore, plant pre-exposed to other diseases also shows increased tolerance to new infecting viruses, a phenomenon referred to as priming (Jung et al., 2009). In natural environments where multiple pathogens are present and mixed infections are prevalent, plant priming could be a common occurrence.

### Impact of Environmental Conditions on Symptom Severity

Environmental conditions such as temperature, light duration and intensity, water availability and concentration of CO_2_ affect viral symptom expression (Hily et al., 2016; Berges et al., 2018). Attenuation of virus-induced symptoms on tobacco plants at extreme temperatures (>36°C or <11°C), called temperature masking, was described almost a century ago (Johnson, 1921; Grainger, 1936). Although, detailed molecular studies in such extreme environments are lacking, the effect of temperature on symptom severity is well-documented in the permissive range (15–30°C). In many cases, temperature-dependent symptom attenuation has been correlated with the regulation of antiviral RNA silencing, as evidenced by the increased accumulation of vsiRNAs at higher temperatures (Szittya et al., 2003). Indeed, plants that are deficient in silencing factors show increased susceptibility to viral infection at higher temperatures (Qu et al., 2005; Zhang et al., 2012; Ghoshal and Sanfacon, 2014). On the other hand, viruses that are deficient in VSR activity can only successfully infect plants at lower temperatures (Szittya et al., 2003). However, the effect of temperature on RNA silencing efficiency can vary with the plant species. SiRNAs are abundantly detected in grapevine plants grown at a range of temperature from 4 to 26°C, but they are not detected in *A. thaliana* plants grown at 4°C (Romon et al., 2013). Indirect effects of temperature on the induction of RNA silencing have also been proposed. Higher temperatures allow more efficient viral RNA replication (Zhang et al., 2012) and this is often associated with earlier symptom development (Obrêpalska-Stêplowska et al., 2015). At lower temperatures, although the initial viral titer is lower, viruses accumulate to higher levels later on and consequently, more severe symptoms can develop at late stages of infection (Szittya et al., 2003; Chellappan et al., 2005; Qu et al., 2005; Ghoshal and Sanfacon, 2014; Xu et al., 2016; Paudel et al., 2018). It is possible that the onset of antiviral RNA silencing, which is triggered when the viral RNA concentration reaches a critical level, occurs earlier at higher temperatures as a consequence of the enhanced virus replication. The efficiency of PTI or ETI is also affected by the growth temperature. In several plant–virus interactions, HR or HR-like responses are slower when the temperature is elevated from 21–22 to 27–28°C and are even prevented at temperatures above 30°C (Whitham et al., 1996; Wang Y. et al., 2009; Jovel et al., 2011). Although increased RNA silencing activity would contribute to temper the expression of defense genes at higher temperatures, it was also shown that the activity and nuclear localization of two R genes (including the *N* gene) are temperature-sensitive directly affecting the defense response (Zhu et al., 2010).

Light intensity also modulates the outcome of plant virus infection. This is not surprising considering that the chloroplast is a major player in plant–virus interactions (Li et al., 2016; Zhao et al., 2016; Bhattacharyya and Chakraborty, 2017). Plants growing under high light conditions show enhanced PTI and ETI responses to various pathogens, including viruses (Chandra-Shekara et al., 2006; Manfre et al., 2011). High light intensity has also been shown to increase localized RNA silencing but reduce the systemic movement of RNA silencing due to shifts in the sink and source status of the leaves (Patil and Fauquet, 2015). Transgenic *N. benthamiana* plants expressing GFP show increased frequency of silencing at higher light intensity and this was correlated with the increased expression of several silencing genes (e.g., *DCL*) (Kotakis et al., 2010, 2011). Consistently, the promoter regions of *DCL* genes contain a light responsive element (Liu et al., 2009).

The level of CO_2_ is another factor that influences plant defenses to pathogen infection (Noctor and Mhamdi, 2017). Growth under high CO_2_ levels triggers the synthesis of SA and primes plant defense responses (Mhamdi and Noctor, 2016). In the context of virus infection, CO_2_ levels have also been shown to influence symptom development (Aguilar et al., 2015; Del Toro et al., 2015; Del Toro et al., 2017). Increased levels of CO_2_ generally result in larger leaf size and can attenuate the impact of virus infection in a virus-specific manner. Higher CO_2_ exposure alleviated some of the negative effects of potato virus Y infection allowing increased accumulation of biomass, nitrogen content and soluble protein but decreased carbon/nitrogen ratio (Ye et al., 2010). Finally, water availability can also impact virus virulence and/or transmission by insect vectors (van Munster et al., 2017; Berges et al., 2018).

The studies described above were conducted in the controlled conditions of experimental greenhouses or growth chambers. However, it is more difficult to predict the impact of the seasonal and diurnal fluctuations of environmental conditions (Sanfacon, 2017; McLeish et al., 2018). Clearly, more studies are warranted to examine plant–virus interactions under field conditions and determine how fluctuating environmental conditions could influence the effectiveness or durability of tolerance.

## Symptom Recovery as an Inducible Form of Tolerance

**FIGURE 2:**
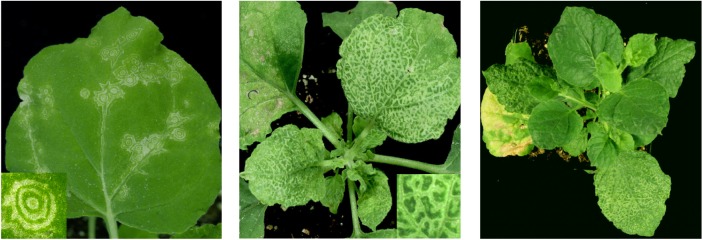
Symptom recovery in *Nicotiana benthamiana* plants infected with tomato ringspot virus. Symptoms are shown during the symptomatic phase of infection as they appear on inoculated leaves **(left)** and systemically infected leaves **(center)**. **(right)** Shows a plant after symptom recovery with asymptomatic young leaves emerging above older symptomatic leaves. Reproduced with permission from Jovel et al. (2007).

Symptom recovery is a typical outcome of some plant–virus interactions, in which plants initially displaying systemic symptoms later recover from infection as exemplified by the emergence of young asymptomatic leaves (Ghoshal and Sanfacon, 2015) (Figure 2). Although the level of viral nucleic acid accumulation is often reduced in recovered leaves (Covey et al., 1997; Szittya et al., 2003; Chellappan et al., 2005; Santovito et al., 2014; Korner et al., 2018), this is not a strict requirement. For example, in the interaction between tomato ringspot virus and *N. benthamiana*, early onset of recovery is not accompanied with a significant reduction of viral RNA levels, although the concentration of viral proteins is reduced (Jovel et al., 2007; Ghoshal and Sanfacon, 2014). Viruses present in recovered tissues maintain their infectivity and protect the plant against secondary infection in a sequence-specific manner (Ratcliff et al., 1997, 1999; Jovel et al., 2007; Santovito et al., 2014; Paudel et al., 2018). This has been attributed to the induction of antiviral RNA silencing during the symptomatic phase of infection (Santovito et al., 2014). Depending on the specific virus, PTGS (viral RNA slicing and/or translation repression), TGS (DNA methylation) or a combination of PTGS and TGS is associated with symptom recovery (Ghoshal and Sanfacon, 2015; Korner et al., 2018). In all cases, the accumulation of viral proteins is reduced to a level below the threshold required for symptom induction. Because young tissues are symptom-free, the host is able to produce seeds. Interestingly, many viruses associated with recovery phenotypes are seed-transmitted. They apparently escape host surveillance mechanisms to invade meristem tissues, at least transiently (reviewed in Ghoshal and Sanfacon, 2015). Thus, symptom recovery can be viewed as an inducible form of tolerance. This makes it an ideal model system for the study of molecular mechanisms associated with tolerance.

## Insights in the Complexity of Tolerant Plant–Virus Interactions Derived From Genetic and Transcriptomic Studies

### Field Tolerance to Virus Infection in Agricultural Crops: Mapping and (Limited) Characterization of Associated Genes

Although tolerance to virus infection is a well-known phenotype in the context of agriculture, the genetic basis for field tolerance is still poorly understood. Genetic crosses and mapping studies have identified a number of quantitative traits loci (QTL) or genes that are associated with tolerance. For example, several genes and QTLs have been linked to tolerance to barley yellow dwarf virus in barley, oat, and wheat (McKenzie et al., 1985; Singh et al., 1993; Jin et al., 1998; Riedel et al., 2011; Del Blanco et al., 2014; Foresman et al., 2016). While in some cases the tolerance was mapped to a single gene, in many cases a combination of major and minor loci were shown to contribute to tolerance and segregation analysis only indicated partial dominance of the major loci. In maize, one to four QTLs were found to be associated with tolerance to maize chlorotic mottle virus in different maize populations (Jones et al., 2017). The QTLs differed with the population, revealing a variety of natural sources for tolerance. In okra, tolerance to yellow vein mosaic virus was mapped to a single dominant gene in two different tolerant cultivars, although other factors were also involved (Senjam et al., 2018). As above, the dominant gene proved to be different in the two cultivars. Tolerance to tomato yellow leaf curl virus is also associated with single dominant genes in wild tomato species and was successfully introgressed into cultivated tomato (Zamir et al., 1994; Vidavsky and Czosnek, 1998). In peach, tolerance to plum pox virus (a potyvirus) was mapped to three loci (Cirilli et al., 2017). One of these loci included a candidate gene with similarities to the *A. thaliana RTM-2* gene, which is implicated in the restriction of the systemic movement of other potyviruses (Cirilli et al., 2017). However, functional validation will be required to confirm whether the *RTM-2*-like gene is indeed responsible for the tolerance. In summary, the variety of dominant, semi-dominant, or recessive tolerance genes found in agricultural crops and the common requirement for multiple loci suggests that molecular mechanisms associated with field tolerance are numerous and complex.

### Host Resource Reallocation in Some but Not All Tolerant Plant–Virus Interactions

Plants can respond to pathogen infection by reallocating resources from vegetative growth to reproduction (i.e., production of seeds). In the *A. thaliana*-cucumber mosaic virus interaction, plants with longer vegetative growth cycles (i.e., longer life spans) are more tolerant to infection (Pagan et al., 2008; Hily et al., 2014; Shukla et al., 2018). Tolerance is also associated with increased seed yield and a shortened reproduction period, reducing the time span between the production of reproductive structures and seed production (Pagan et al., 2008). However, *A. thaliana* that were tolerant to cucumber mosaic virus did not show similar resource reallocation in response to more virulent viruses, suggesting that this response is virus specific (Shukla et al., 2018). In addition, tolerant plants with extended vegetative growth resulting from resource allocation were less competitive in the context of dense plant populations (Pagan et al., 2009). Additional studies using a variety of tolerant plant–virus interactions grown under various environmental conditions should shed more light on the biological relevance of resource allocation. Little is known regarding underlying molecular mechanism associated with resource reallocation. However, it is likely that they would require multiple genetic determinants affecting various regulatory mechanisms that control plant growth and development.

### Reprogramming of the Plant Transcriptome in Tolerant Interactions Affecting Defense Pathways, Primary Metabolism, and Hormone Signaling

Virus infection induces global changes in the plant transcriptome and proteome in both susceptible and resistant interactions (Palukaitis et al., 2008; Llave, 2016). To date, only a limited number of transcriptomic studies have focussed on tolerant interactions (reviewed in Bengyella et al., 2015). Transcriptome changes have been characterized at different stages of virus infection in a tolerant interaction (Stare et al., 2015). Time-course studies have also allowed monitoring symptomatic and asymptomatic phases of infection associated with symptom recovery or with delayed symptom induction (Allie et al., 2014; Madronero et al., 2018). Finally, transcriptomes or proteomes have been compared in susceptible, resistant or tolerant cultivars infected with the same virus strain (Allie et al., 2014; Wang et al., 2016b) or in plants infected with virulent or mild virus strains (Kogovsek et al., 2016; Geng et al., 2017). Not surprisingly, these studies have highlighted both similarities and differences in the transcriptome changes induced by viruses in susceptible, tolerant, and resistant interactions. In many cases, similar plant pathways are affected in the different types of interactions but to different extents or with different dynamics. Pathways commonly impacted by virus infection include defense responses (e.g., R-like genes and PR proteins), primary metabolism, photosynthesis, and hormone signaling.

In the interaction between potato virus Y and the tolerant potato cultivar Désirée, photosynthesis genes were shown to be transiently induced at early stages of infection but then rapidly repressed at the onset of virus multiplication (Stare et al., 2015). It was suggested that the early induction of photosynthesis (and other primary metabolism associated genes) helps promote the induction of defense responses. Transgenic Désirée, transformed with the *NahG* gene that down-regulates SA signaling, showed more severe symptoms upon virus infection and a diminished induction of photosynthesis genes at early stages of infection (Stare et al., 2015). Analysis of small RNA signaling in the potato virus Y-potato cv. Désirée interaction revealed induction of miRNAs known to down-regulate R-like genes and the presence of vsiRNAs that target plant stress signaling response genes. Plant small RNAs that down-regulate the gibberellin synthesis were also induced and this affected the levels of miR482f, a key regulator of R-like gene expression (Kriznik et al., 2017). This complex regulation of small RNA pathways was shown to be dependent on SA signaling.

Other studies have also shown increased induction of SA signaling, defense response proteins or R-like genes in tolerant cultivars or in asymptomatic phases of infection compared to corresponding symptomatic interactions (Sahu et al., 2012; Allie et al., 2014; Louis and Rey, 2015; Wang et al., 2016b; Madronero et al., 2018). Many of these studies also noted altered primary metabolism. In some cases, increased expression of antiviral RNA silencing genes was also observed in tolerant interactions (Sahu et al., 2012; Allie et al., 2014). The impact of JA and Et signaling pathways is less clear. Delayed symptom induction in the interaction between papaya and the papaya meleira virus complex is associated with concomitant induction of both SA-defense responses and the antagonistic JA pathway (Madronero et al., 2018). Similarly, although susceptible cassava cultivars show reduced JA and Et signaling after infection with South African cassava mosaic virus, a tolerant cultivar does not (Allie et al., 2014). Taken together these studies highlight the complex regulatory networks between various plant hormone signaling pathways and defense responses.

Although the analysis of global transcriptome changes provides useful insights in the intricacy of plant–virus interactions, it is not always clear whether these changes are the cause or consequence of tolerance. Also, since transcriptomic studies do not examine post-transcriptional changes in gene expression, it is not known whether changes in the transcriptome are also reflected in the plant proteome. In fact, a recent study highlighted major discrepancies between transcriptomic and proteomic data that may be of biological significance (Stare et al., 2017). In addition, environmental factors are also predicted to impact the outcome of transcriptome studies. Indeed, transcriptomics analysis of plants exposed under combination of three different stresses exhibit significant differences in their gene expression compared to plants exposed under single stress (Prasch and Sonnewald, 2013). These issues are exemplified in a recent analysis of the expression of AGO2 in plants grown at two temperatures and infected with two tomato ringspot virus isolates of varying virulence (Paudel et al., 2018). Although AGO2 mRNAs were transiently induced to similar levels under all conditions, the accumulation of the AGO2 protein was influenced by the isolate and the growth temperature. Plants that later recovered from infection showed increased accumulation of AGO2 protein at early stages of infection. However, mutation of AGO2 did not prevent the symptom recovery suggesting that other factors influence the outcome of infection.

## Molecular Mechanisms Associated With Achieving a Balance Between Antiviral RNA Silencing and Virus Counter-Defense Responses

As described above, symptom recovery, an inducible form of tolerance, is associated with the induction of antiviral RNA silencing. Thus, it could be assumed that viruses that are associated with symptom recovery phenotypes do not suppress silencing efficiently. In fact, mutation of potent VSRs from virulent viruses can lead to symptom recovery (reviewed in Ghoshal and Sanfacon, 2015). On the other hand, ectopic expression of potent VSRs (e.g., the potyvirus HC-Pro) can prevent symptom recovery in nepovirus-infected plants (Siddiqui et al., 2008; Santovito et al., 2014). However, viruses that encode strong VSRs can also be associated with symptom recovery, as long as the activity of these VSRs is reduced in recovered leaves as recently shown in *A. thaliana* plants infected with oilseed rape mosaic virus (Korner et al., 2018). Thus, suppression of antiviral RNA silencing occurs during the initial stages to allow systemic viral infection, and symptom recovery depends on achieving a balance between antiviral RNA silencing and VSR activity during the recovery stage.

Some viruses deploy self-attenuation mechanisms to achieve this balance. Indeed, some viral proteins function to attenuate the accumulation and/or activity of VSRs. Symptom recovery is the normal outcome of the interaction between an isolate of cucumber mosaic virus and *A. thaliana*. However, symptoms were exacerbated by mutation of an Arg-rich region of the CP (Zhang et al., 2017). The wild-type CP was shown to attenuate the silencing suppression activity of the VSR (the 2b protein). This is probably achieved by inhibiting the translation of 2b, via the RNA-binding activity of the CP (Zhang et al., 2017). It was also proposed that binding of the CP to the viral RNA may protect it from degradation and allow enhanced production of vsiRNAs (Zhang et al., 2017), although this will need to be confirmed experimentally.

Another example of viral self-attenuation is provided by the plum pox virus-*N. benthamiana* pathosystem. Plum pox virus proteins are initially expressed as a single large polyprotein (Revers and Garcia, 2015). The P1 protease is the N-terminal protein domain in the polyprotein. Cleavage by P1 contributes to the release of the VSR (HC-Pro, the second protein domain) from the polyprotein. Because the HC-Pro silencing suppression activity is impaired by fusion to P1, the efficiency of the P1 proteolytic cleavage directly affects the activity of HC-Pro (Pasin et al., 2014). Deletion of the N-terminal region of P1 accelerated the release of HC-Pro from the polyprotein, enhanced its VSR activity, stimulated initial accumulation of the virus and enhanced the induction of the HR necrotic response, contributing to the enhanced symptomatology (Pasin et al., 2014). It was suggested that the N-terminal region of P1 interacts with a host factor to down-regulate the P1 proteolytic activity. The N-terminal region of the P1 protein is highly variable and later work confirmed that it is involved in host adaptation (Shan et al., 2015, 2017). It was hypothesized that the N-terminal region of the P1 protein, although dispensable, is maintained to prevent virus over-accumulation (Pasin et al., 2014).

Strikingly, a viral protein was also shown to enhance the plant antiviral RNA silencing. Viral RNAs move cell-to-cell by modifying the natural channels between plant cells (the plasmodesmata), creating a virus front that invades naïve cells (reviewed in Harries and Ding, 2011; Heinlein, 2015). The vsiRNAs follow a similar route, moving through the plasmodesmata. Intriguingly, the tobacco mosaic virus MP was shown to facilitate the movement of vsiRNAs, thus functioning in a manner opposite to that of many characterized VSRs that hinder vsiRNAs movement (Vogler et al., 2008). Thus, while tobacco mosaic virus encodes a potent VSR to suppress anti-viral silencing, this activity is apparently counter-balanced by that of the MP. Since the MP is only expressed transiently early in infection, this self-attenuation effect would likely also only be effective in the critical initial stages of infection, i.e., at the front of infection (Vogler et al., 2008; Amari et al., 2012). On the other hand, enhancing vsiRNAs movement may also render naïve cells more susceptible to the incoming virus by down-regulating specific plant genes that are targeted by these vsiRNAs (Amari et al., 2012).

Defective-interfering RNAs (diRNAs) are associated with several viruses and have been shown to attenuate symptoms induced by the parent virus. The diRNAs contain non-contiguous segments from the parent viral RNA and are produced by template-switching of the viral RNA-dependent RNA polymerase (RdRp) during viral RNA replication (Simon et al., 2004; Pathak and Nagy, 2009). They contain all the *cis-*acting elements necessary for their continued replication by the viral RdRp and can accumulate *de novo* to very high levels. They interfere with the replication of the parent viral RNAs and prevent over-accumulation of viral products. The mechanisms of diRNA interference are not completely understood. While the *cis-*acting elements present on diRNAs may out-compete the viral RNAs for the viral RdRp and for host factors, other mechanisms likely also play a role, including the enhancement of antiviral RNA silencing (Simon et al., 2004; Pathak and Nagy, 2009). In tombusvirus infected-plants, diRNAs are recognized by DCL enzymes, leading to the enhanced synthesis of siRNAs that share sequences with the parent viral RNAs (Havelda et al., 2005). As described above, the tombusvirus p19 VSR functions by binding to vsiRNAs and sequestering them away from RISC complexes (Scholthof, 2006). However, the binding capacity of p19 was shown to be saturated in the presence of diRNAs leading to increased antiviral RNA silencing against the parental virus (Havelda et al., 2005). Interestingly, a second silencing suppression activity of p19 is not affected by the presence of diRNAs, suggesting that the VSR and the diRNAs act in an antagonistic manner to regulate the levels of virus accumulation in infected plants (Varallyay et al., 2014). Indeed, p19 induces the synthesis of miR168, which down-regulates the expression of AGO1, one of the main effectors of antiviral RNA silencing. The induction of miR168 by p19 was found to be similar in the presence or absence of diRNAs (Varallyay et al., 2014).

Additional evidence for antagonistic interactions between VSRs and diRNAs is documented for the interaction between a crinivirus and *N. benthamiana* (Lukhovitskaya et al., 2013). The 8K viral protein is a weak VSR that enhances virus accumulation. Interestingly, the coding region for the 8K protein was implicated in the template-switching mechanism that produces the diRNAs. It was suggested that diRNAs are essential regulatory molecules that minimize the impact of crinivirus infection on their hosts (Lukhovitskaya et al., 2013). While the role of diRNAs in symptom attenuation is well-established in model hosts under laboratory conditions, their impact on infections in the field or in natural environments is not well-studied and clearly deserves further investigation.

## Molecular Mechanisms Aimed at Limiting the Accumulation or Activity of Viral Proteins

Plants may be able to accommodate substantial levels of viral nucleic acid accumulation without significant damage, as long as they manage the concentration or activities of viral proteins that orchestrate interactions with plant factors and act as virulence factors (Culver and Padmanabhan, 2007). As will be described below, this can be achieved by repressing the translation of viral RNAs, by destabilizing viral proteins or by modulating their activity.

### Repression of Viral Genome Translation

Translation repression has emerged as a common mechanism of RNA silencing-mediated gene regulation in plants (Brodersen et al., 2008; Iwakawa and Tomari, 2013) and has also been suggested to operate against plant viruses in association with tolerance or with symptom recovery phenotypes. In *N. benthamiana* plants infected with tomato ringspot virus, the initial stages of symptom recovery are associated with a drastic reduction in viral protein levels but not with a concomitant reduction in viral RNA concentration (Jovel et al., 2007; Ghoshal and Sanfacon, 2014). Translation of viral RNA2 was shown to be repressed at the onset of symptom recovery and silencing of AGO1 prevented both the translation repression and the symptom recovery (Ghoshal and Sanfacon, 2014). Similarly, recovery of *A. thaliana* from oilseed rape mosaic virus was shown to be dependent on AGO1 and was associated with translation repression preventing over-accumulation of the VSR (Korner et al., 2018). Finally, the reduction of viral titers in late stages of the asymptomatic infection of *A. thaliana* plants with tobacco rattle virus was concomitant with a decrease in ribosome-associated viral RNAs and an increase in the number of processing bodies (Ma et al., 2015), which are RNA granules often associated with translation repression mechanism (Makinen et al., 2017). Although these studies suggest a role for antiviral RNA silencing translation repression mechanisms in tolerant interactions, a direct role for AGO-containing RISC complexes in the translation repression of viral RNAs has not been experimentally confirmed.

A distinct translation repression mechanism is directed by a transmembrane receptor, NIK1 (NSP-interacting kinase), which is related to leucine-rich repeat receptor-like kinases implicated in the innate immune PTI response (Machado et al., 2015). NIK1 was first identified as an interactor of begomovirus NSP1 protein. NIK1 also interacts with and phosphorylates ribosomal protein RPL10A, redirecting this protein to the nucleus (Carvalho et al., 2008). Once in the nucleus, RPL10A interacts with L10-INTERACTING MYB DOMAIN CONTAINING PROTEIN (LIMYB), a transcription factor that regulates the expression of ribosomal genes (Zorzatto et al., 2015). The RLP10A-LIMYB interaction causes massive down-regulation of ribosomal genes and global translation repression, which also impairs virus translation. Importantly, the translation repression is specifically induced upon virus infection and depends on the autophosphorylation of NIK1 at tyrosine 474. Knock-out of the *NIK1*, *RPL10A*, or *LIMYB* genes exacerbates symptoms and enhances virus accumulation, confirming the importance of the translation repression mechanism in limiting virus-induced damage to the plant (Carvalho et al., 2008; Zorzatto et al., 2015). As a counter-defense, the viral NSP protein suppresses the activity of NIK1 preventing its autophosphorylation (Fontes et al., 2004). Interestingly, ectopic expression of a phosphomimic mutant of AtNIK1 with a mutation of tyrosine 474 to aspartic acid, bypassed the counter-defense and provided broad-spectrum tolerance to begomoviruses in tomato, with minimal impact on plant growth in non-infected plants (Brustolini et al., 2015).

In addition to the plant responses described above, viruses minimize the accumulation of viral virulence factors (e.g., VSRs, RdRps) using sub-optimal translation initiation codons or inefficient frameshift or read-through translation mechanisms (reviewed in Miras et al., 2017). These are highly conserved features of viral genomes, highlighting their importance for viral self-attenuation mechanisms.

### Using Cellular Protein Degradation Pathways to Prevent Over-Accumulation of Viral Proteins and to Regulate Plant Defense Responses

Cellular protein degradation mechanisms, in particular the ubiquitin/26S proteasome system (UPS) and the autophagy pathway are key regulators of plant–virus interactions (Alcaide-Loridan and Jupin, 2012; Verchot, 2016b; Clavel et al., 2017; Ustun et al., 2017). By controlling the accumulation of viral and/or plant proteins, they modulate plant defense responses, regulate viral counter-defense responses, control the viral infection cycle and mitigate symptoms. It could be argued that both partners in the interaction benefit from manipulating protein degradation pathways. Indeed, that viral proteins maintain conserved signature sequences for recognition by plant degradation pathways could be viewed as evidence for virus self-attenuation.

Protein substrates targeted by the UPS are ubiquitinated at lysine residues by cellular E3 ubiquitin-ligases, a large family of plant proteins (1400 genes in *A. thaliana*). Depending on the nature of the ubiquitination (mono- or poly-ubiquitination), proteins are selectively targeted to the 26S proteasome for degradation. Cellular E3 ubiquitin ligases are common interactors of plant virus proteins, including, MPs and RdRps, many of which are destabilized by the UPS (Alcaide-Loridan and Jupin, 2012; Verchot, 2016b). Turnip yellow mosaic virus RdRp contains a highly-conserved PEST sequence, which is recognized as a degradation trigger (Camborde et al., 2010). Interestingly, the viral protease acts as a deubiquitinase to protect the RdRp from UPS degradation (Chenon et al., 2012). These results suggest that a delicate cross-talk between viral enzymes and the plant UPS regulates the accumulation of the viral RdRp.

Direct evidence for a role for the UPS in facilitating tolerance is exemplified in the interaction between *N. benthamiana* and tomato yellow leaf curl China virus (TYLCCV) (Shen et al., 2016). The TYLCCV-associated betasatellite DNA encodes βC1, a symptom determinant and a VSR. βC1 interacts with NtRFP1, a plant RING E3 ligase and is targeted to degradation by the 26S proteasome. βC1 induces severe stunting and leaf curling symptoms when over-expressed in transgenic lines (Yang et al., 2008). However, in natural infection it only accumulates to low levels, and symptoms are milder. Symptoms are further attenuated in plants overexpressing NtRFP1, while plants knocked-down for NtRFP1 develop more severe symptoms (Shen et al., 2016). Importantly, viral DNA accumulation is not affected by manipulation of NtRFP1 expression. Thus, this study demonstrates how the destabilization of a viral pathogenicity factor by the UPS can mitigate symptom expression while allowing systemic virus infection. A separate study demonstrated an interaction between cotton leaf curl Multan virus (CLCuMuV) βC1 protein and a distinct E3 ligase complex (the SCF complex) (Jia et al., 2016). However, the CLCuMuV βC1 protein was shown to inhibit the SCF E3 ligase, allowing enhanced virus accumulation and more severe symptoms. These apparently conflicting results are not necessarily mutually exclusive. Rather, they highlight the complexity of the interactions between plant viruses and various branches of the UPS pathway.

Autophagy is another highly conserved protein degradation pathway implicated in many aspects of plant–pathogens interactions including the regulation of programmed cell death (Ustun et al., 2017). Proteins targeted by the autophagy pathway are directed to double-membrane vesicles, autophagosomes, before they are finally released in the vacuoles for degradation. There are extensive cross-talks between autophagy and the UPS degradation pathways. For example the AUTOPHAGY-RELATED GENE 6 (ATG6) protein is ubiquitinated by SINAT E3 ligases and degraded by the 26S proteasome (Qi et al., 2017). Therefore, it is perhaps not surprising that the CLCuMuV βC1 protein is not only interacting with UPS components, but it is also targeted for degradation by the autophagy pathway following its interaction with ATG8 (Haxim et al., 2017). Preventing the interaction between βC1 and ATG8 exacerbated symptoms and enhanced virus accumulation. Similarly, silencing of ATG5 and ATG7 increased the plant susceptibility to three geminiviruses. These results highlight a role for autophagy in mitigating the impact of geminivirus infection. Similarly, other VSRs are also degraded through the autophagy pathway, notably the potyvirus HC-Pro protein and the cucumovirus 2b protein (Nakahara et al., 2012). This requires an interaction between the VSRs and rgsCaM, a calmudolin-like protein and an endogenous suppressor of silencing, which is itself destined to autophagic degradation. Interestingly, rgsCaM is also a component of the SA-mediated systemic acquired resistance (Jeon et al., 2017).

Another interesting example of regulated autophagic protein degradation comes from the interaction between cauliflower mosaic virus and *A. thaliana* (Hafren et al., 2017). The viral CP interacts with NEIGHBOR OF BRCA1 (NBR1), an autophagy receptor and is targeted to autophagic degradation. This limits virus accumulation early in infection. Later on, virus particles accumulate in inclusion bodies, where they are protected from autophagy (Hafren et al., 2017). The CaMV P6 protein, which represses SA-mediated autophagy, may also help relieve the CP degradation (Zvereva et al., 2016). Similarly, NBR1 is required for the autophagic degradation of the turnip mosaic virus HC-Pro but this is counteracted by two other viral proteins (Hafren et al., 2018). Thus, viral proteins have evolved to be susceptible to degradation by the autophagy pathway and protected from this degradation at different stages of infection. In addition, induction of the autophagy prevented early cell death in these two pathosystems. Indeed, *A. thaliana* mutants deficient in the autophagy pathway display more severe symptoms than wild-type plants after infection with either turnip mosaic virus or cauliflower mosaic virus in a manner that is independent of the level of viral accumulation (Hafren et al., 2017, 2018). Inhibition of SA-mediated autophagy by the CaMV P6 protein also contributes to symptom severity. P6 activates the TOR (target of rapamycin) kinase, a down-regulator of autophagy and exacerbates symptoms, which are normally mitigated by the autophagy pathway (Zvereva et al., 2016). Transgenic lines that express the P6 protein from severe CaMV isolates display chlorotic and dwarfing symptoms, while those expressing the P6 protein from a mild isolate do not (Yu et al., 2003). Interestingly, the P6 protein from this mild isolate is unable to activate TOR or disrupt SA-mediated autophagy (Zvereva et al., 2016).

Finally, the UPS and autophagy pathways are usurped by viruses to target plant defense proteins. A case in point is the ability of several VSRs to target plant RNA silencing factors (notably AGO proteins) to degradation (Csorba et al., 2015). Thus, plant protein degradation pathways modulate both the plant antiviral defenses and the virus counter-defenses.

### Regulating the Activity of Viral Proteins With Post-translational Modifications

Another approach to mitigate the impact of toxic viral proteins is to control their activity. This can be achieved by post-translational modification. For example, phosphorylation of the βC1 protein from the betasatellite DNA of TYLCCV by the SNF1-related protein kinase 1 (SnRK1) reduces its silencing suppression activity and diminishes symptom severity (Zhong et al., 2017). Similarly, phosphorylation of the cabbage leaf curl virus VSR (the AL2 protein) delays the symptom formation in *A. thaliana* (Shen et al., 2014). In the case of the turnip yellow mosaic virus RdRp, phosphorylation of the conserved PEST sequence is a prerequisite for its subsequent destabilization by the plant proteasome degradation pathway (Jakubiec et al., 2006). On the other hand, phosphorylation has also been shown to be required for the function of viral CPs, MPs, or RdRps (Stork et al., 2005; Champagne et al., 2007; Kleinow et al., 2009). While the role of protein modification in the regulation of plant–virus interactions is still poorly understood, especially in the context of tolerance, its importance cannot be underestimated.

## Molecular Mechanisms Deployed to Relieve Virus-Induced Stress of the Plant Endoplasmic Reticulum

As mentioned above, virus infection commonly causes ER stress, which needs to be relieved to prevent cell death (Zhang and Wang, 2012; Verchot, 2016a). In plants, ER stress is sensed by transmembrane proteins [e.g., the inositol requiring enzyme (IRE1) and the Bax inhibitor 1 (BI-1) proteins] that induce the unfolded protein response (UPR) to restore proper protein folding in the ER and prevent aggregation. Activation of IRE1 causes splicing of the bZIP60 transcription factor transcript and production of a truncated form of the transcription factor, which translocate to the nucleus to induce the expression of UPR-related genes, including calcium-dependent protein chaperones (e.g., Bip, calmudolin, calreticulin). The ER-associated degradation (ERAD) pathway is also activated as part of the UPR. ERAD functions by translocating unfolded or misfolded proteins back into the cytoplasm where they are degraded by the cytosolic UPS or autophagic pathways. Evidence for the importance of the UPR in mitigating the consequences of virus-induced ER stress is accumulating. Expression of viral integral membrane proteins has been reported to induce the UPR (Ye et al., 2011, 2013; Zhang L. et al., 2015; Gaguancela et al., 2016). For example, the expression of IRE1 and BI-1 is induced by the potexvirus TGB3 or potyvirus 6K2 integral membrane proteins (Gaguancela et al., 2016). Down-regulation of BI-1 or bZIP60 in *N. benthamiana* allowed increased systemic accumulation of potato virus X and potato virus Y and exacerbated systemic necrosis symptoms indicating that the UPR is induced to release ER stress, control virus accumulation, and prevent cell death (Gaguancela et al., 2016). Consistently, overexpression of the ER Bip chaperone suppresses TGB3-induced cell death in *N. benthamiana* infected with potato virus X (Ye et al., 2011, 2013). Intriguingly, down-regulation of IRE1/bZIP60 has also been shown to hinder accumulation of turnip mosaic virus, *in A. thaliana* and to ameliorate non-necrotic virus-induced symptoms, suggesting that in this interaction the UPR actually promotes virus infection, possibly also by mitigating the consequences of ER stress (Zhang L. et al., 2015). Other plant–virus interactions will need to be examined before we can obtain a more complete understanding of the role of the UPR in facilitating tolerant plant–virus interactions. Finally, how the ERAD and downstream protein degradation pathways contribute to mitigating virus-induced ER stress is also not well-characterized.

## Tolerance Conferred by Mutation of an Ineffective R-Like Gene to Prevent Systemic Lethal Necrosis

The *A. thaliana TTR1* semi-dominant locus was shown to determine symptom expression following infection with tobacco ringspot virus (Lee et al., 1996). Screening of 97 *A. thaliana* lines revealed that although the virus accumulated to similar levels, the intensity of symptoms varied greatly. Systemic necrosis killed the most susceptible lines while tolerant plants were either asymptomatic or only displayed mild symptoms. The *TTR1* gene present in susceptible lines was later shown to correspond to a R-like gene (Nam et al., 2011). An HR-like response was activated in plants with the *TTR1* gene, but the replication and movement of the virus were not restricted and systemic acquired resistance was not established. It was suggested that the systemic lethal necrosis phenotype was caused by a runaway HR response. Interestingly, transfer of the *TTR1* gene to *N. benthamiana* also caused lethal systemic necrosis. The tolerant phenotype in *A. thaliana* accessions was found to be associated with mutations of the *TTR1* gene which prevented the establishment of the systemic HR response (Nam et al., 2011).

## Engineering Tolerance to Virus Disease for Field Application: Current Progress and Future Opportunities

As highlighted above, tolerance is a complex genetic trait that involves multiple molecular mechanisms operating simultaneously, many of which are yet to be discovered. The benefits of tolerance compared to resistance have also been described in terms of reduced selection pressure for the emergence of virulent isolates, increased breadth and stability of the phenotype and potential benefits to the host (as exemplified in natural environments). Although natural sources of tolerance are available for some economically important crops, they are generally poorly characterized and have been of limited use. The next question becomes: is it feasible to engineer tolerance for practical field applications?

Only a few examples of engineered tolerance to virus diseases can be found in the literature. Most relate to the identification and manipulation of plant genes involved in signal transduction pathways associated with basal innate immune defense responses. Some are broad-spectrum and also provide tolerance to abiotic stress, in part because some of the signaling pathways are overlapping. We have already discussed how the ectopic expression of a phosphomimic mutant of AtNIK1, an immune receptor kinase, conferred broad-spectrum tolerance to begomovirus infection in tomato (Brustolini et al., 2015). Other kinases implicated in defense signal transduction pathways have also been manipulated to mitigate viral symptoms. Overexpression of SlMAPK3, a MAP kinase, showed enhanced expression of defense genes associated with SA- and JA-signaling, lower accumulation of reactive oxygen species, increased accumulation of antioxidant enzymes, and stronger tolerance to tomato yellow leaf curl virus infection as expressed by a 2-week delay in symptom induction which was sufficient to allow plant flowering (Li et al., 2017). Similarly, overexpression of OsCIPK30, a kinase involved in calcium signaling, in rice provided enhance tolerance to rice stripe virus, that was associated with delayed and milder symptoms and enhanced expression of PR genes (Liu et al., 2017).

Overexpression of a transcription factor, the soybean GmERF3 gene, in tobacco also conferred increased tolerance to tobacco mosaic virus (Zhang et al., 2009). This transcription factor is induced in response to various stresses and up-regulates the expression of many defense genes, including PR proteins. Thus, overexpression of this gene activated the plant basal immunity, achieving a result similar to the plant transcriptome reprogramming observed in several natural tolerant interactions. The tolerance level was modest resulting in delay in the establishment of symptoms rather than long-term symptom attenuation. Increased tolerance to salt, drought, and fungal diseases was also achieved after overexpression of this gene (Zhang et al., 2009).

As discussed above, manipulation of plant genes implicated in protein degradation pathways or the UPR response may also provide novel avenues to engineer tolerance. Examples include the overexpression of the NtRFP1 RING E3 ligase to promote tolerance in *N. benthamiana* plants infected with a begomovirus (Shen et al., 2016), or of the ER Bip chaperone to suppress cell death associated with potexvirus infection of *N. benthamiana* (Ye et al., 2013). Down-regulation of genes associated with the UPR response has been shown to reduce symptom expression in other plant–virus interactions (Zhang L. et al., 2015).

The study of highly symptomatic interactions can help identify novel sources of tolerance. For example, a transcriptomic study of a systemic symptomatic infection associated with runaway HR necrosis conferred by a soybean R gene in response to a virulent isolate of soybean mosaic virus identified eIF5A as a highly induced gene (Chen et al., 2017). eIF5A is a translation factor previously implicated in symptom development in the interaction between *A. thaliana* and the bacterium *Pseudomonas syringae*. Interestingly, silencing of this gene diminished the systemic necrosis and reduced virus accumulation (Chen et al., 2017).

Although tolerance can been enhanced by manipulating plant signaling pathways in herbaceous hosts under controlled environmental conditions, the feasibility of field applications needs to be examined. Indeed, modifying vital plant signaling pathways is likely to have pleiotropic effects that could vary depending on each plant–virus interaction and could also impact tolerance to other biotic or abiotic stresses. In addition, even if experiments conducted with herbaceous hosts under limited time periods show minimal impact on the plant growth and development, plants with longer lifespans (for example trees) could be affected differently. Further research aimed at elucidating the molecular mechanisms associated with tolerance, in particular in wild plant–virus interactions, may identify novel targets for engineering tolerance or assist in the development of improved agriculture practices.

## Author Contributions

DP and HS jointly wrote the manuscript, and read and approved the final manuscript.

## Conflict of Interest Statement

The authors declare that the research was conducted in the absence of any commercial or financial relationships that could be construed as a potential conflict of interest.

## References

[B1] Aguilar E. Allende L. Del Toro F. J. Chung B. N. Canto T. Tenllado F. (2015). Effects of elevated CO(2)and temperature on pathogenicity determinants and virulence of potato virus X/*Potyvirus*-associated synergism. *Mol. Plant Microbe Interact.* 28 1364–1373. 10.1094/MPMI-08-15-0178-R 26422405

[B2] Aguilar E. Cutrona C. Del Toro F. J. (2017). Virulence determines beneficial trade-offs in the response of virus-infected plants to drought via induction of salicylic acid. *Plant Cell Environ.* 40 2909–2930. 10.1111/pce.13028 28718885

[B3] Alazem M. Lin N. S. (2015). Roles of plant hormones in the regulation of host-virus interactions. *Mol. Plant Pathol.* 16 529–540. 10.1111/mpp.12204 25220680 PMC6638471

[B4] Alcaide-Loridan C. Jupin I. (2012). Ubiquitin and plant viruses, Let’s play together! *Plant Physiol.* 160 72–82. 22802610 10.1104/pp.112.201905 PMC3440231

[B5] Allie F. Pierce E. J. Okoniewski M. J. Rey C. (2014). Transcriptional analysis of South African cassava mosaic virus-infected susceptible and tolerant landraces of cassava highlights differences in resistance, basal defense and cell wall associated genes during infection. *BMC Genomics* 15:1006. 10.1186/1471-2164-15-1006 25412561 PMC4253015

[B6] Amari K. Vazquez F. Heinlein M. (2012). Manipulation of plant host susceptibility: an emerging role for viral movement proteins? *Front. Plant Sci.* 3:10. 10.3389/fpls.2012.00010 22639637 PMC3355624

[B7] Anandalakshmi R. Pruss G. J. Ge X. (1998). A viral suppressor of gene silencing in plants. *Proc. Natl. Acad. Sci. U.S.A.* 95 13079–13084. 9789044 10.1073/pnas.95.22.13079 PMC23715

[B8] Bazzini A. A. Almasia N. I. Manacorda C. A. (2009). Virus infection elevates transcriptional activity of miR164a promoter in plants. *BMC Plant Biol.* 9:152. 10.1186/1471-2229-9-152 20042107 PMC2809068

[B9] Bengyella L. Waikhom S. D. Allie F. Rey C. (2015). Virus tolerance and recovery from viral induced-symptoms in plants are associated with transcriptome reprograming. *Plant Mol. Biol.* 89 243–252. 10.1007/s11103-015-0362-6 26358043

[B10] Berges S. E. Vile D. Vazquez-Rovere C. (2018). Interactions between drought and plant genotype change epidemiological traits of *Cauliflower mosaic virus*. *Front. Plant Sci.* 9:703. 10.3389/fpls.2018.00703 29881396 PMC5976794

[B11] Bhattacharyya D. Chakraborty S. (2017). Chloroplast: the trojan horse in plant-virus interaction. *Mol. Plant Pathol.* 19 504–518. 10.1111/mpp.12533 28056496 PMC6638057

[B12] Bigeard J. Colcombet J. Hirt H. (2015). Signaling mechanisms in pattern-triggered immunity (PTI). *Mol. Plant* 8 521–539. 10.1016/j.molp.2014.12.022 25744358

[B13] Bilgin D. D. Zavala J. A. Zhu J. Clough S. J. Ort D. R. DeLucia E. H. (2010). Biotic stress globally downregulates photosynthesis genes. *Plant Cell Environ.* 33 1597–1613. 10.1111/j.1365-3040.2010.02167.x 20444224

[B14] Blanc S. Michalakis Y. (2016). Manipulation of hosts and vectors by plant viruses and impact of the environment. *Curr. Opin. Insect Sci.* 16 36–43. 10.1016/j.cois.2016.05.007 27720048

[B15] Boccara M. Sarazin A. Thiebeauld O. (2014). The *Arabidopsis* miR472-RDR6 silencing pathway modulates PAMP- and effector-triggered immunity through the post-transcriptional control of disease resistance genes. *PLoS Pathog.* 10:e1003883. 10.1371/journal.ppat.1003883 24453975 PMC3894208

[B16] Bolouri Moghaddam M. R. Van den Ende W. (2012). Sugars and plant innate immunity. *J. Exp. Bot.* 63 3989–3998. 10.1093/jxb/ers129 22553288

[B17] Bolton M. D. (2009). Primary metabolism and plant defense–fuel for the fire. *Mol. Plant Microbe Interact.* 22 487–497. 10.1094/MPMI-22-5-0487 19348567

[B18] Borges F. Martienssen R. A. (2015). The expanding world of small RNAs in plants. *Nat. Rev. Mol. Cell Biol.* 16 727–741. 10.1038/nrm4085 26530390 PMC4948178

[B19] Bortolamiol D. Pazhouhandeh M. Marrocco K. Genschik P. Ziegler-Graff V. (2007). The polerovirus F box protein P0 targets ARGONAUTE1 to suppress RNA silencing. *Curr. Biol.* 17 1615–1621. 17869109 10.1016/j.cub.2007.07.061

[B20] Brodersen P. Sakvarelidze-Achard L. Bruun-Rasmussen M. (2008). Widespread translational inhibition by plant miRNAs and siRNAs. *Science* 320 1185–1190. 10.1126/science.1159151 18483398

[B21] Brustolini O. J. Machado J. P. Condori-Apfata J. A. (2015). Sustained NIK-mediated antiviral signalling confers broad-spectrum tolerance to begomoviruses in cultivated plants. *Plant Biotechnol. J.* 13 1300–1311. 10.1111/pbi.12349 25688422 PMC4857726

[B22] Camborde L. Planchais S. Tournier V. (2010). The ubiquitin-proteasome system regulates the accumulation of Turnip yellow mosaic virus RNA-dependent RNA polymerase during viral infection. *Plant Cell* 22 3142–3152. 10.1105/tpc.109.072090 20823192 PMC2965540

[B23] Carr J. P. Murphy A. M. Tungadi T. Yoon J.-Y. (2018). Plant defense signals: players and pawns in plant-virus-vector interactions. *Plant Sci.* (in press) 10.1016/j.plantsci.2018.04.011 30709497

[B24] Carvalho C. M. Santos A. A. Pires S. R. (2008). Regulated nuclear trafficking of rpL10A mediated by NIK1 represents a defense strategy of plant cells against virus. *PLoS Pathog.* 4:e1000247. 10.1371/journal.ppat.1000247 19112492 PMC2597721

[B25] Champagne J. Laliberte-Gagne M. E. Leclerc D. (2007). Phosphorylation of the termini of *Cauliflower mosaic virus* precapsid protein is important for productive infection. *Mol. Plant Microbe Interact.* 20 648–658. 17555273 10.1094/MPMI-20-6-0648

[B26] Chandra-Shekara A. C. Gupte M. Navarre D. (2006). Light-dependent hypersensitive response and resistance signaling against *Turnip crinkle virus* in *Arabidopsis*. *Plant J.* 45 320–334. 16412080 10.1111/j.1365-313X.2005.02618.x

[B27] Chapman E. J. Prokhnevsky A. I. Gopinath K. Dolja V. V. Carrington J. C. (2004). Viral RNA silencing suppressors inhibit the microRNA pathway at an intermediate step. *Genes Dev.* 18 1179–1186. 15131083 10.1101/gad.1201204 PMC415642

[B28] Chellappan P. Vanitharani R. Ogbe F. Fauquet C. M. (2005). Effect of temperature on geminivirus-induced RNA silencing in plants. *Plant Physiol.* 138 1828–1841. 16040661 10.1104/pp.105.066563 PMC1183375

[B29] Chen H. Adam Arsovski A. Yu K. Wang A. (2017). Deep sequencing leads to the identification of eukaryotic translation initiation factor 5A as a key element in Rsv1-mediated lethal systemic hypersensitive response to *Soybean mosaic virus* infection in soybean. *Mol. Plant Pathol.* 18 391–404. 10.1111/mpp.12407 27019403 PMC6638201

[B30] Chenon M. Camborde L. Cheminant S. Jupin I. (2012). A viral deubiquitylating enzyme targets viral RNA-dependent RNA polymerase and affects viral infectivity. *EMBO J.* 31 741–753. 10.1038/emboj.2011.424 22117220 PMC3273391

[B31] Cirilli M. Rossini L. Geuna F. (2017). Genetic dissection of Sharka disease tolerance in peach (*P. persica* L. Batsch). *BMC Plant Biol.* 17:192. 10.1186/s12870-017-1117-0 29100531 PMC5670703

[B32] Clavel M. Michaeli S. Genschik P. (2017). Autophagy: a double-edged sword to fight plant viruses. *Trends Plant Sci.* 22 646–648. 10.1016/j.tplants.2017.06.007 28633985

[B33] Cooper J. I. Jones A. T. (1983). Responses of plants to viruses: proposals for the use of terms. *Phytopathology* 73 127–128.

[B34] Covey S. N. Al-Kaff N. S. Langara A. Turner D. S. (1997). Plants combat infection by gene silencing. *Nature* 385 781–782.

[B35] Csorba T. Kontra L. Burgyan J. (2015). Viral silencing suppressors: tools forged to fine-tune host-pathogen coexistence. *Virology* 479-480 85–103. 10.1016/j.virol.2015.02.028 25766638

[B36] Cui J. You C. Chen X. (2017). The evolution of microRNAs in plants. *Curr. Opin. Plant Biol.* 35 61–67. 10.1016/j.pbi.2016.11.006 27886593 PMC5342909

[B37] Culver J. N. Padmanabhan M. S. (2007). Virus-induced disease: altering host physiology one interaction at a time. *Annu. Rev. Phytopathol.* 45 221–243. 17417941 10.1146/annurev.phyto.45.062806.094422

[B38] Dastogeer K. M. G. Li H. Sivasithamparam K. Jones M. G. K. Wylie S. J. (2018). Fungal endophytes and a virus confer drought tolerance to *Nicotiana benthamiana* plants through modulating osmolytes, antioxidant enzymes and expression of host drought responsive genes. *Environ. Exp. Bot.* 149 95–108.

[B39] de Ronde D. Butterbach P. Kormelink R. (2014). Dominant resistance against plant viruses. *Front. Plant Sci.* 5:307. 10.3389/fpls.2014.00307 25018765 PMC4073217

[B40] Del Blanco I. A. Hegarty J. Gallagher L. (2014). Mapping of QTL for tolerance to cereal yellow dwarf virus in two-rowed spring barley. *Crop Sci.* 54 1468–1475. 10.2135/cropsci2013.11.0781 27212713 PMC4874343

[B41] Del Toro F. J. Aguilar E. Hernandez-Walias F. J. Tenllado F. Chung B. N. Canto T. (2015). High temperature, high ambient CO_2_ affect the interactions between three positive-sense RNA viruses and a compatible host differentially, but not their silencing suppression efficiencies. *PLoS One* 10:e0136062. 10.1371/journal.pone.0136062 PMC4551900 26313753

[B42] Del Toro F. J. Rakhshandehroo F. Larruy B. Aguilar E. Tenllado F. Canto T. (2017). Effects of simultaneously elevated temperature and CO_2_ levels on *Nicotiana benthamiana* and its infection by different positive-sense RNA viruses are cumulative and virus type-specific. *Virology* 511 184–192. 10.1016/j.virol.2017.08.015 28866237

[B43] Dempsey D. A. Vlot A. C. Wildermuth M. C. Klessig D. F. (2011). Salicylic acid biosynthesis and metabolism. *Arabidopsis Book* 9:e0156. 10.1199/tab.0156 22303280 PMC3268552

[B44] Deng Y. Wang J. Tung J. (2018). A role for small RNA in regulating innate immunity during plant growth. *PLoS Pathog.* 14:e1006756. 10.1371/journal.ppat.1006756 29293695 PMC5766230

[B45] Donze T. Qu F. Twigg P. Morris T. J. (2014). *Turnip crinkle virus* coat protein inhibits the basal immune response to virus invasion in *Arabidopsis* by binding to the NAC transcription factor TIP. *Virology* 449 207–214. 10.1016/j.virol.2013.11.018 24418554 PMC3906646

[B46] Endres M. W. Gregory B. D. Gao Z. (2010). Two plant viral suppressors of silencing require the ethylene-inducible host transcription factor RAV2 to block RNA silencing. *PLoS Pathog.* 6:e1000729. 10.1371/journal.ppat.1000729 20084269 PMC2800190

[B47] Fontes E. P. Santos A. A. Luz D. F. Waclawovsky A. J. Chory J. (2004). The geminivirus nuclear shuttle protein is a virulence factor that suppresses transmembrane receptor kinase activity. *Genes Dev.* 18 2545–2556. 15489295 10.1101/gad.1245904 PMC529541

[B48] Foresman B. J. Oliver R. E. Jackson E. W. Chao S. Arruda M. P. Kolb F. L. (2016). Genome-wide association mapping of *Barley yellow dwarf virus* tolerance in spring oat (*Avena sativa* L.). *PLoS One* 11:e0155376. 10.1371/journal.pone.0155376 27175781 PMC4866777

[B49] Gaguancela O. A. Zuniga L. P. Arias A. V. (2016). The IRE1/bZIP60 pathway and bax inhibitor 1 suppress systemic accumulation of *Potyviruses* and *Potexviruses* in *Arabidopsis* and *Nicotiana benthamiana* plants. *Mol. Plant Microbe Interact.* 29 750–766. 27578623 10.1094/MPMI-07-16-0147-R

[B50] García-Arenal F. Fraile A. (2013). Trade-offs in host range evolution of plant viruses. *Plant Pathol.* 62 2–9.

[B51] Geng C. Wang H. Y. Liu J. (2017). Transcriptomic changes in *Nicotiana benthamiana* plants inoculated with the wild-type or an attenuated mutant of Tobacco vein banding mosaic virus. *Mol. Plant Pathol.* 18 1175–1188. 10.1111/mpp.12471 27539720 PMC6638280

[B52] Geri C. Love A. J. Cecchini E. (2004). *Arabidopsis* mutants that suppress the phenotype induced by transgene-mediated expression of *Cauliflower mosaic virus* (CaMV) gene VI are less susceptible to CaMV-infection and show reduced ethylene sensitivity. *Plant Mol. Biol.* 56 111–124. 15604731 10.1007/s11103-004-2649-x

[B53] Ghoshal B. Sanfacon H. (2014). Temperature-dependent symptom recovery in *Nicotiana benthamiana* plants infected with *Tomato ringspot virus* is associated with reduced translation of viral RNA2 and requires ARGONAUTE 1. *Virology* 45 188–197. 10.1016/j.virol.2014.03.026 24889238

[B54] Ghoshal B. Sanfacon H. (2015). Symptom recovery in virus-infected plants: revisiting the role of RNA silencing mechanisms. *Virology* 47 167–179. 10.1016/j.virol.2015.01.008 25677651

[B55] Gouveia B. C. Calil I. P. Machado J. P. B. Santos A. A. Fontes E. P. B. (2017). Immune receptors and Co-receptors in antiviral innate immunity in plants. *Front. Microbiol.* 7:2139. 10.3389/fmicb.2016.02139 28105028 PMC5214455

[B56] Grainger J. (1936). Low-temperature masking of *Tobacco mosaic* symptoms. *Nature* 137 31–32.

[B57] Guo Q. Major I. T. Howe G. A. (2018). Resolution of growth–defense conflict: mechanistic insights from jasmonate signaling. *Curr. Opin. Plant Biol.* 44 72–81. 10.1016/j.pbi.2018.02.009 29555489

[B58] Hafren A. Macia J. L. Love A. J. Milner J. J. Drucker M. Hofius D. (2017). Selective autophagy limits *Cauliflower mosaic virus* infection by NBR1-mediated targeting of viral capsid protein and particles. *Proc. Natl. Acad. Sci. U.S.A.* 114 E2026–E2035. 10.1073/pnas.1610687114 28223514 PMC5347569

[B59] Hafren A. Ustun S. Hochmuth A. Svenning S. Johansen T. Hofius D. (2018). *Turnip mosaic virus* counteracts selective autophagy of the viral silencing suppressor HCpro. *Plant Physiol.* 176 649–662. 10.1104/pp.17.01198 29133371 PMC5761789

[B60] Hamelin F. M. Hilker F. M. Sun T. A. (2017). The evolution of parasitic and mutualistic plant-virus symbioses through transmission-virulence trade-offs. *Virus Res.* 241 77–87. 10.1016/j.virusres.2017.04.011 28434906

[B61] Harries P. Ding B. (2011). Cellular factors in plant virus movement: at the leading edge of macromolecular trafficking in plants. *Virology* 411 237–243. 10.1016/j.virol.2010.12.021 21239029

[B62] Hashimoto M. Neriya Y. Yamaji Y. Namba S. (2016). Recessive resistance to plant viruses: potential resistance genes beyond translation initiation factors. *Front. Microbiol.* 7:1695. 10.3389/fmicb.2016.01695 27833593 PMC5080351

[B63] Havelda Z. Hornyik C. Valoczi A. Burgyan J. (2005). Defective interfering RNA hinders the activity of a *Tombusvirus*-encoded posttranscriptional gene silencing suppressor. *J. Virol.* 79 450–457. 15596838 10.1128/JVI.79.1.450-457.2005 PMC538711

[B64] Havko N. E. Major I. T. Jewell J. B. Attaran E. Browse J. Howe G. A. (2016). Control of carbon assimilation and partitioning by jasmonate: an accounting of growth-defense tradeoffs. *Plants* 5:E7. 10.3390/plants5010007 27135227 PMC4844420

[B65] Haxim Y. Ismayil A. Jia Q. (2017). Autophagy functions as an antiviral mechanism against geminiviruses in plants. *eLife* 6:e23897. 10.7554/eLife.23897 28244873 PMC5362266

[B66] He X. F. Fang Y. Y. Feng L. Guo H. S. (2008). Characterization of conserved and novel microRNAs and their targets, including a TuMV-induced TIR-NBS-LRR class R gene-derived novel miRNA in *Brassica*. *FEBS Lett.* 582 2445–2452. 10.1016/j.febslet.2008.06.011 18558089

[B67] Heil M. Baldwin I. T. (2002). Fitness costs of induced resistance: emerging experimental support for a slippery concept. *Trends Plant Sci.* 7 61–67. 11832276 10.1016/s1360-1385(01)02186-0

[B68] Heinlein M. (2015). Plant virus replication and movement. *Virology* 479–480, 657–671. 10.1016/j.virol.2015.01.025 25746797

[B69] Hily J. M. Garcia A. Moreno A. (2014). The relationship between host lifespan and pathogen reservoir potential: an analysis in the system *Arabidopsis thaliana*-*Cucumber mosaic virus*. *PLoS Pathog.* 10:e1004492. 10.1371/journal.ppat.1004492 25375140 PMC4223077

[B70] Hily J. M. Poulicard N. Mora M. A. Pagan I. Garcia-Arenal F. (2016). Environment and host genotype determine the outcome of a plant-virus interaction: from antagonism to mutualism. *New Phytol.* 209 812–822. 10.1111/nph.13631 26365599

[B71] Huot B. Yao J. Montgomery B. L. He S. Y. (2014). Growth–defense tradeoffs in plants: a balancing act to optimize fitness. *Mol. Plant* 7 1267–1287. 10.1093/mp/ssu049 24777989 PMC4168297

[B72] Hussain M. Mansoor S. Iram S. Zafar Y. Briddon R. W. (2007). The hypersensitive response to tomato leaf curl new delhi virus nuclear shuttle protein is inhibited by transcriptional activator protein. *Mol. Plant Microbe Interact.* 20 1581–1588. 17990965 10.1094/MPMI-20-12-1581

[B73] Ishibashi K. Ishikawa M. (2014). Mechanisms of tomato mosaic virus RNA replication and its inhibition by the host resistance factor Tm-1. *Curr. Opin. Virol.* 9 8–13. 10.1016/j.coviro.2014.08.005 25212767

[B74] Iwakawa H. O. Tomari Y. (2013). Molecular insights into microRNA-mediated translational repression in plants. *Mol. Cell.* 52 591–601. 10.1016/j.molcel.2013.10.033 24267452

[B75] Jackel J. N. Buchmann R. C. Singhal U. Bisaro D. M. (2015). Analysis of geminivirus AL2 and L2 proteins reveals a novel AL2 silencing suppressor activity. *J. Virol.* 89 3176–3187. 10.1128/JVI.02625-14 25552721 PMC4337558

[B76] Jakubiec A. Tournier V. Drugeon G. (2006). Phosphorylation of viral RNA-dependent RNA polymerase and its role in replication of a plus-strand RNA virus. *J. Biol. Chem.* 281 21236–21249. 16717096 10.1074/jbc.M600052200

[B77] Jeon E. J. Tadamura K. Murakami T. (2017). rgs-CaM detects and counteracts viral RNA silencing suppressors in plant immune priming. *J. Virol.* 91:e00761-17. 10.1128/JVI.00761-17 28724770 PMC5599751

[B78] Ji L. H. Ding S. W. (2001). The suppressor of transgene RNA silencing encoded by *Cucumber mosaic virus* interferes with salicylic acid-mediated virus resistance. *Mol. Plant Microbe Interact.* 14 715–724. 11386367 10.1094/MPMI.2001.14.6.715

[B79] Jia Q. Liu N. Xie K. (2016). CLCuMuB betaC1 subverts ubiquitination by interacting with NbSKP1s to enhance Geminivirus infection in *Nicotiana benthamiana*. *PLoS Pathog.* 12:e1005668. 10.1371/journal.ppat.1005668 27315204 PMC4912122

[B80] Jin H. Domier L. L. Kolb F. L. Brown C. M. (1998). Identification of quantitative Loci for tolerance to *Barley yellow dwarf virus* in oat. *Phytopathology* 88 410–415. 10.1094/PHYTO.1998.88.5.410 18944919

[B81] Jin X. Cao X. Wang X. (2018). Three-dimensional architecture and biogenesis of membrane structures associated with plant virus replication. *Front. Plant Sci.* 9:57. 10.3389/fpls.2018.00057 29441085 PMC5797596

[B82] Johnson J. (1921). The relation of air temperature to certain plant diseases. *Phytopathology* 11 446–458. 10.1097/MOO.0b013e3283524b14 22569402

[B83] Jones J. D. Dangl J. L. (2006). The plant immune system. *Nature* 444 323–329. 17108957 10.1038/nature05286

[B84] Jones M. W. Penning B. W. Jamann T. M. (2017). Diverse chromosomal locations of quantitative trait loci for tolerance to maize chlorotic mottle virus in five maize populations. *Phytopathology* 108 748–758. 10.1094/PHYTO-09-17-0321-R 29287150

[B85] Jovel J. Walker M. Sanfacon H. (2007). Recovery of *Nicotiana benthamiana* plants from a necrotic response induced by a nepovirus is associated with RNA silencing but not with reduced virus titer. *J. Virol.* 81 12285–12297. 17728227 10.1128/JVI.01192-07 PMC2168989

[B86] Jovel J. Walker M. Sanfacon H. (2011). Salicylic acid-dependent restriction of *Tomato ringspot virus* spread in tobacco is accompanied by a hypersensitive response, local rna silencing, and moderate systemic resistance. *Mol. Plant Microbe Interact.* 24 706–718. 10.1094/MPMI-09-10-0224 21281112

[B87] Jung H. W. Tschaplinski T. J. Wang L. Glazebrook J. Greenberg J. T. (2009). Priming in systemic plant immunity. *Science* 324 89–91. 10.1126/science.1170025 19342588

[B88] Kangasjarvi S. Neukermans J. Li S. Aro E. M. Noctor G. (2012). Photosynthesis, photorespiration, and light signalling in defence responses. *J. Exp. Bot.* 63 1619–1636. 10.1093/jxb/err402 22282535

[B89] Kleinow T. Nischang M. Beck A. (2009). Three C-terminal phosphorylation sites in the *Abutilon mosaic virus* movement protein affect symptom development and viral DNA accumulation. *Virology* 390 89–101. 10.1016/j.virol.2009.04.018 19464722

[B90] Kogovsek P. Pompe-Novak M. Petek M. Fragner L. Weckwerth W. Gruden K. (2016). Primary metabolism, phenylpropanoids and antioxidant pathways are regulated in potato as a response to *Potato virus Y* infection. *PLoS One* 11:e0146135. 10.1371/journal.pone.0146135 26727123 PMC4738437

[B91] Kontra L. Csorba T. Tavazza M. (2016). Distinct effects of p19 RNA silencing suppressor on small RNA mediated pathways in plants. *PLoS Pathog.* 12:e1005935. 10.1371/journal.ppat.1005935 27711201 PMC5053613

[B92] Korbecka-Glinka G. Czubacka A. Przybys M. Doroszewska T. (2017). Resistance vs. tolerance to *Potato virus Y* in tobacco-comparing effectiveness using virus isolates from Central Europe. *Breed. Sci.* 67 459–465. 10.1270/jsbbs.17019 29398939 PMC5790041

[B93] Korner C. J. Pitzalis N. Pena E. J. Erhardt M. Vazquez F. Heinlein M. (2018). Crosstalk between PTGS and TGS pathways in natural antiviral immunity and disease recovery. *Nat. Plants* 4 157–164. 10.1038/s41477-018-0117-x 29497161

[B94] Kotakis C. Vrettos N. Daskalaki M. G. Kotzabasis K. Kalantidis K. (2011). DCL3 and DCL4 are likely involved in the light intensity-RNA silencing cross talk in *Nicotiana benthamiana*. *Plant Signal. Behav.* 6 1180–1182. 10.4161/psb.6.8.15689 21791977 PMC3260716

[B95] Kotakis C. Vrettos N. Kotsis D. Tsagris M. Kotzabasis K. Kalantidis K. (2010). Light intensity affects RNA silencing of a transgene in *Nicotiana benthamiana* plants. *BMC Plant Biol.* 10:220. 10.1186/1471-2229-10-220 20939918 PMC3017829

[B96] Kriznik M. Petek M. Dobnik D. (2017). Salicylic acid perturbs sRNA-Gibberellin regulatory network in immune response of potato to *Potato virus Y* infection. *Front. Plant Sci.* 8:2192. 10.3389/fpls.2017.02192 29312421 PMC5744193

[B97] Künstler A. Bacsó R. Gullner G. Hafez Y. M. Király L. (2016). Staying alive – is cell death dispensable for plant disease resistance during the hypersensitive response? *Physiol. Mol. Plant Pathol.* 93 75–84.

[B98] Laliberte J. F. Sanfacon H. (2010). Cellular remodeling during plant virus infection. *Annu. Rev. Phytopathol.* 48 69–91. 10.1146/annurev-phyto-073009-114239 20337516

[B99] Lee J. M. Hartman G. L. Domier L. L. Bent A. F. (1996). Identification and map location of TTR1, a single locus in *Arabidopsis thaliana* that confers tolerance to tobacco ringspot nepovirus. *Mol. Plant Microbe Interact.* 9 729–735. 8870272 10.1094/mpmi-9-0729

[B100] Li F. Pignatta D. Bendix C. (2012). MicroRNA regulation of plant innate immune receptors. *Proc. Natl. Acad. Sci. U.S.A.* 109 1790–1795. 10.1073/pnas.1118282109 22307647 PMC3277104

[B101] Li H. W. Lucy A. P. Guo H. S. (1999). Strong host resistance targeted against a viral suppressor of the plant gene silencing defence mechanism. *EMBO J.* 18 2683–2691. 10329615 10.1093/emboj/18.10.2683 PMC1171350

[B102] Li Y. Cui H. Cui X. Wang A. (2016). The altered photosynthetic machinery during compatible virus infection. *Curr. Opin. Virol.* 17 19–24. 10.1016/j.coviro.2015.11.002 26651024

[B103] Li Y. Qin L. Zhao J. (2017). SlMAPK3 enhances tolerance to *Tomato yellow leaf curl virus* (TYLCV) by regulating salicylic acid and jasmonic acid signaling in tomato (*Solanum lycopersicum*). *PLoS One* 12:e0172466. 10.1371/journal.pone.0172466 28222174 PMC5319765

[B104] Liu Q. Feng Y. Zhu Z. (2009). Dicer-like (DCL) proteins in plants. *Funct. Integr. Genomics* 9 277–286. 10.1007/s10142-009-0111-5 19221817

[B105] Liu Z. Li X. Sun F. Zhou T. Zhou Y. (2017). Overexpression of OsCIPK30 enhances plant tolerance to *Rice stripe virus*. *Front. Microbiol.* 8:2322. 10.3389/fmicb.2017.02322 29225594 PMC5705616

[B106] Llave C. (2016). Dynamic cross-talk between host primary metabolism and viruses during infections in plants. *Curr. Opin. Virol.* 19 50–55. 10.1016/j.coviro.2016.06.013 27442236

[B107] Louis B. Rey C. (2015). Resistance gene analogs involved in tolerant cassava–geminivirus interaction that shows a recovery phenotype. *Virus Genes* 51 393–407. 10.1007/s11262-015-1246-1 26370397

[B108] Love A. J. Geri C. Laird J. (2012). *Cauliflower mosaic virus* protein P6 inhibits signaling responses to salicylic acid and regulates innate immunity. *PLoS One* 7:e47535. 10.1371/journal.pone.0047535 23071821 PMC3469532

[B109] Lozano-Duran R. Rosas-Diaz T. Gusmaroli G. (2011). Geminiviruses subvert ubiquitination by altering CSN-mediated derubylation of SCF E3 ligase complexes and inhibit jasmonate signaling in *Arabidopsis thaliana*. *Plant Cell* 23 1014–1032. 10.1105/tpc.110.080267 21441437 PMC3082251

[B110] Lu R. Folimonov A. Shintaku M. (2004). Three distinct suppressors of RNA silencing encoded by a 20-kb viral RNA genome. *Proc. Natl. Acad. Sci. U.S.A.* 101 15742–15747. 15505219 10.1073/pnas.0404940101 PMC524217

[B111] Lukan T. Baebler S. Pompe-Novak M. (2018). Cell death is not sufficient for the restriction of *Potato Virus Y* spread in hypersensitive response-conferred resistance in potato. *Front. Plant Sci.* 9:168. 10.3389/fpls.2018.00168 29497431 PMC5818463

[B112] Lukhovitskaya N. I. Thaduri S. Garushyants S. K. Torrance L. Savenkov E. I. (2013). Deciphering the mechanism of defective interfering RNA (DI RNA) biogenesis reveals that a viral protein and the DI RNA Act antagonistically in virus infection. *J. Virol.* 87 6091–6103. 10.1128/JVI.03322-12 23514891 PMC3648117

[B113] Ma X. Nicole M. C. Meteignier L. V. Hong N. Wang G. Moffett P. (2015). Different roles for RNA silencing and RNA processing components in virus recovery and virus-induced gene silencing in plants. *J. Exp. Bot.* 66 919–932. 10.1093/jxb/eru447 25385769

[B114] Machado J. P. Brustolini O. J. Mendes G. C. Santos A. A. Fontes E. P. (2015). NIK1, a host factor specialized in antiviral defense or a novel general regulator of plant immunity? *Bioessays* 37 1236–1242. 10.1002/bies.201500066 26335701

[B115] Madronero J. Rodrigues S. P. Antunes T. F. S. (2018). Transcriptome analysis provides insights into the delayed sticky disease symptoms in *Carica papaya*. *Plant Cell Rep.* 37 967–980. 10.1007/s00299-018-2281-x 29564545

[B116] Makinen K. Lohmus A. Pollari M. (2017). Plant RNA regulatory network and RNA granules in virus infection. *Front. Plant Sci.* 8:2093. 10.3389/fpls.2017.02093 29312371 PMC5732267

[B117] Mandadi K. K. Scholthof K. B. (2013). Plant immune responses against viruses: how does a virus cause disease? *Plant Cell* 25 1489–1505. 10.1105/tpc.113.111658 23709626 PMC3694688

[B118] Manfre A. Glenn M. Nunez A. Moreau R. A. Dardick C. (2011). Light quantity and photosystem function mediate host susceptibility to *Turnip mosaic virus* via a salicylic acid-independent mechanism. *Mol. Plant Microbe Interact.* 24 315–327. 10.1094/MPMI-08-10-0191 21091158

[B119] Martinez de Alba A. E. Elvira-Matelot E. Vaucheret H. (2013). Gene silencing in plants: a diversity of pathways. *Biochim. Biophys. Acta* 1829 1300–1308. 10.1016/j.bbagrm.2013.10.005 24185199

[B120] Mascia T. Gallitelli D. (2016). Synergies and antagonisms in virus interactions. *Plant Sci.* 252 176–192. 10.1016/j.plantsci.2016.07.015 27717453

[B121] McKenzie R. I. H. Burnett P. A. Gill C. C. Comeau A. Brown P. D. (1985). Inheritance of tolerance to *Barley yellow dwarf virus* in oats. *Euphytica* 34 681–687.

[B122] McLeish M. J. Fraile A. Garcia-Arenal F. (2018). Ecological complexity in plant virus host range evolution. *Adv. Virus Res.* 101 293–339. 10.1016/bs.aivir.2018.02.009 29908592

[B123] Mhamdi A. Noctor G. (2016). High CO_2_ primes plant biotic stress defences through redox-linked pathways. *Plant Physiol.* 172 929–942. 27578552 10.1104/pp.16.01129 PMC5047113

[B124] Miozzi L. Gambino G. Burgyan J. Pantaleo V. (2013). Genome-wide identification of viral and host transcripts targeted by viral siRNAs in *Vitis vinifera*. *Mol. Plant Pathol.* 14 30–43. 10.1111/j.1364-3703.2012.00828.x 22947170 PMC6638717

[B125] Miras M. Miller W. A. Truniger V. Aranda M. A. (2017). Non-canonical translation in plant RNA viruses. *Front. Plant Sci.* 8:494. 10.3389/fpls.2017.00494 28428795 PMC5382211

[B126] Miyashita S. Takahashi H. (2015). R-gene-mediated resistance to plant viruses. *Uirusu* 65 199–208. 27760918 10.2222/jsv.65.199

[B127] Miyashita Y. Atsumi G. Nakahara K. S. (2016). Trade-offs for viruses in overcoming innate immunities in plants. *Mol. Plant Microbe Interact.* 29 595–598. 10.1094/MPMI-05-16-0103-CR 27294885

[B128] Moffett P. (2009). “Mechanisms of recognition in dominant R gene mediated resistance,” in *Natural and Engineered Resistance to Plant Viruses, Part I*, ed. Loebenstein G. (Amsterdam: Elsevier Science).

[B129] Moon J. Y. Park J. M. (2016). Cross-talk in viral defense signaling in plants. *Front. Microbiol.* 7:2068 10.3389/fmicb.2016.02068 PMC5174109 28066385

[B130] Moyo L. Ramesh S. V. Kappagantu M. Mitter N. Sathuvalli V. Pappu H. R. (2017). The effects of potato virus Y-derived virus small interfering RNAs of three biologically distinct strains on potato (*Solanum tuberosum*) transcriptome. *Virol. J.* 14:129. 10.1186/s12985-017-0803-8 28716126 PMC5513076

[B131] Mubin M. Amin I. Amrao L. Briddon R. W. Mansoor S. (2010). The hypersensitive response induced by the V2 protein of a monopartite begomovirus is countered by the C2 protein. *Mol. Plant Pathol.* 11 245–254. 10.1111/j.1364-3703.2009.00601.x 20447273 PMC6640282

[B132] Nagy P. D. (2016). *Tombusvirus*-host interactions: co-opted evolutionarily conserved host factors take center court. *Annu. Rev. Virol.* 3 491–515. 27578441 10.1146/annurev-virology-110615-042312

[B133] Nagy P. D. Pogany J. (2012). The dependence of viral RNA replication on co-opted host factors. *Nat. Rev. Microbiol.* 10 137–149. 10.1038/nrmicro2692 22183253 PMC7097227

[B134] Nakahara K. S. Masuta C. Yamada S. (2012). Tobacco calmodulin-like protein provides secondary defense by binding to and directing degradation of virus RNA silencing suppressors. *Proc. Natl. Acad. Sci. U.S.A.* 109 10113–10118. 10.1073/pnas.1201628109 22665793 PMC3382489

[B135] Nam M. Koh S. Kim S. U. (2011). *Arabidopsis* TTR1 causes LRR-dependent lethal systemic necrosis, rather than systemic acquired resistance, to *Tobacco ringspot virus*. *Mol. Cells* 32 421–429. 10.1007/s10059-011-0101-z 22057987 PMC3887690

[B136] Nicaise V. (2017). Boosting innate immunity to sustainably control diseases in crops. *Curr. Opin. Virol.* 26 112–119. 10.1016/j.coviro.2017.07.030 28802707

[B137] Nicaise V. Candresse T. (2017). Plum pox virus capsid protein suppresses plant pathogen-associated molecular pattern (PAMP)-triggered immunity. *Mol. Plant Pathol.* 18 878–886. 10.1111/mpp.12447 27301551 PMC6638313

[B138] Niehl A. Pena E. J. Amari K. Heinlein M. (2013). Microtubules in viral replication and transport. *Plant J.* 75 290–308. 23379770 10.1111/tpj.12134

[B139] Niehl A. Wyrsch I. Boller T. Heinlein M. (2016). Double-stranded RNAs induce a pattern-triggered immune signaling pathway in plants. *New Phytol.* 211 1008–1019. 10.1111/nph.13944 27030513

[B140] Nieva J. L. Madan V. Carrasco L. (2012). Viroporins: structure and biological functions. *Nat. Rev. Microbiol.* 10 563–574. 10.1038/nrmicro2820 22751485 PMC7097105

[B141] Noctor G. Mhamdi A. (2017). Climate change, CO_2_, and defense: the metabolic, redox, and signaling perspectives. *Trends Plant Sci.* 22 857–870. 10.1016/j.tplants.2017.07.007 28811163

[B142] Obrêpalska-Stêplowska A. Renaut J. Planchon S. (2015). Effect of temperature on the pathogenesis, accumulation of viral and satellite RNAs and on plant proteome in peanut stunt virus and satellite RNA-infected plants. *Front. Plant Sci.* 6:903. 10.3389/fpls.2015.00903 26579153 PMC4625170

[B143] Pagan I. Alonso-Blanco C. Garcia-Arenal F. (2007). The relationship of within-host multiplication and virulence in a plant-virus system. *PLoS One* 2:e786. 10.1371/journal.pone.0000786 17726516 PMC1950075

[B144] Pagan I. Alonso-Blanco C. Garcia-Arenal F. (2008). Host responses in life-history traits and tolerance to virus infection in *Arabidopsis thaliana*. *PLoS Pathog.* 4:e1000124. 10.1371/journal.ppat.1000124 18704166 PMC2494869

[B145] Pagan I. Alonso-Blanco C. Garcia-Arenal F. (2009). Differential tolerance to direct and indirect density-dependent costs of viral infection in *Arabidopsis thaliana*. *PLoS Pathog.* 5:e1000531. 10.1371/journal.ppat.1000531 19649316 PMC2712083

[B146] Pagan I. Garcia-Arenal F. (2018). Tolerance to plant pathogens: theory and experimental evidence. *Int. J. Mol. Sci.* 19:E810. 10.3390/ijms19030810 29534493 PMC5877671

[B147] Pallas V. Garcia J. A. (2011). How do plant viruses induce disease? Interactions and interference with host components. *J. Gen. Virol.* 92 2691–2705. 10.1099/vir.0.034603-0 21900418

[B148] Palukaitis P. Carr J. P. Schoelz J. E. (2008). Plant-virus interactions. *Methods Mol. Biol.* 451 3–19. 10.1007/978-1-59745-102-4_1 18370244

[B149] Pasin F. Simon-Mateo C. Garcia J. A. (2014). The hypervariable amino-terminus of P1 protease modulates potyviral replication and host defense responses. *PLoS Pathog.* 10:e1003985. 10.1371/journal.ppat.1003985 24603811 PMC3946448

[B150] Pathak K. B. Nagy P. D. (2009). Defective interfering RNAs: foes of viruses and friends of virologists. *Viruses* 1 895–919. 10.3390/v1030895 21994575 PMC3185524

[B151] Patil B. L. Fauquet C. M. (2015). Light intensity and temperature affect systemic spread of silencing signal in transient agroinfiltration studies. *Mol. Plant Pathol.* 16 484–494. 10.1111/mpp.12205 25220764 PMC6638542

[B152] Paudel D. B. Ghoshal B. Jossey S. Ludman M. Fatyol K. Sanfacon H. (2018). Expression and antiviral function of ARGONAUTE 2 in *Nicotiana benthamiana* plants infected with two isolates of *Tomato ringspot virus* with varying degrees of virulence. *Virology* 524 127–139. 10.1016/j.virol.2018.08.016 30195250

[B153] Pertermann R. Tamilarasan S. Gursinsky T. (2018). A Viral suppressor modulates the plant immune response early in infection by regulating MicroRNA activity. *mBio* 9:e00419-18. 10.1128/mBio.00419-18 29691336 PMC5915741

[B154] Pitzalis N. Heinlein M. (2017). The roles of membranes and associated cytoskeleton in plant virus replication and cell-to-cell movement. *J. Exp. Bot.* 69 117–132. 10.1093/jxb/erx334 29036578

[B155] Poque S. Wu H. W. Huang C. H. (2018). Potyviral Gene-silencing suppressor HCPro interacts with Salicylic Acid (SA)-binding protein 3 to weaken SA-mediated defense responses. *Mol. Plant Microbe Interact.* 31 86–100. 10.1094/MPMI-06-17-0128-FI 29090655

[B156] Prasch C. M. Sonnewald U. (2013). Simultaneous application of heat, drought, and virus to *Arabidopsis* plants reveals significant shifts in signaling networks. *Plant Physiol.* 162 1849–1866. 10.1104/pp.113.221044 23753177 PMC3729766

[B157] Pruss G. J. Lawrence C. B. Bass T. Li Q. Q. Bowman L. H. Vance V. (2004). The potyviral suppressor of RNA silencing confers enhanced resistance to multiple pathogens. *Virology* 320 107–120. 15003867 10.1016/j.virol.2003.11.027

[B158] Qi H. Xia F. N. Xie L. J. (2017). TRAF family proteins regulate autophagy dynamics by modulating autophagy protein6 stability in *Arabidopsis*. *Plant Cell* 29 890–911. 10.1105/tpc.17.00056 28351989 PMC5435438

[B159] Qi X. Bao F. S. Xie Z. (2009). Small RNA deep sequencing reveals role for *Arabidopsis thaliana* RNA-dependent RNA polymerases in viral siRNA biogenesis. *PLoS One* 4:e4971. 10.1371/journal.pone.0004971 19308254 PMC2654919

[B160] Qu F. Ye X. Hou G. Sato S. Clemente T. E. Morris T. J. (2005). RDR6 has a broad-spectrum but temperature-dependent antiviral defense role in *Nicotiana benthamiana*. *J. Virol.* 79 15209–15217. 16306592 10.1128/JVI.79.24.15209-15217.2005 PMC1316014

[B161] Raja P. Wolf J. N. Bisaro D. M. (2010). RNA silencing directed against geminiviruses: post-transcriptional and epigenetic components. *Biochim. Biophys. Acta* 1799 337–351. 10.1016/j.bbagrm.2010.01.004 20079472

[B162] Ramesh S. V. Sahu P. P. Prasad M. Praveen S. Pappu H. R. (2017). Geminiviruses and plant hosts: a closer examination of the molecular arms race. *Viruses* 9:E256. 10.3390/v9090256 28914771 PMC5618022

[B163] Ratcliff F. Harrison B. D. Baulcombe D. C. (1997). A similarity between viral defense and gene silencing in plants. *Science* 276 1558–1560. 18610513 10.1126/science.276.5318.1558

[B164] Ratcliff F. G. MacFarlane S. A. Baulcombe D. C. (1999). Gene silencing without DNA. RNA-mediated cross-protection between viruses. *Plant Cell* 11 1207–1216. 10402423 10.1105/tpc.11.7.1207 PMC144281

[B165] Rausher M. D. (2001). Co-evolution and plant resistance to natural enemies. *Nature* 411 857–864. 11459070 10.1038/35081193

[B166] Revers F. Garcia J. A. (2015). Molecular biology of *Potyviruses*. *Adv. Virus Res.* 92 101–199. 10.1016/bs.aivir.2014.11.006 25701887

[B167] Riedel C. Habekuss A. Schliephake E. Niks R. Broer I. Ordon F. (2011). Pyramiding of Ryd2 and Ryd3 conferring tolerance to a German isolate of *Barley yellow dwarf virus*-PAV (BYDV-PAV-ASL-1) leads to quantitative resistance against this isolate. *Theor. Appl. Genet.* 123 69–76. 10.1007/s00122-011-1567-y 21416402

[B168] Robert-Seilaniantz A. Grant M. Jones J. D. (2011). Hormone crosstalk in plant disease and defense: more than just Jasmonate-Salicylate antagonism. *Annu. Rev. Phytopathol.* 49 317–343. 10.1146/annurev-phyto-073009-114447 21663438

[B169] Rojas C. M. Senthil-Kumar M. Tzin V. Mysore K. S. (2014). Regulation of primary plant metabolism during plant-pathogen interactions and its contribution to plant defense. *Front. Plant Sci.* 5:17. 10.3389/fpls.2014.00017 24575102 PMC3919437

[B170] Romon M. Soustre-Gacougnolle I. Schmitt C. (2013). RNA silencing is resistant to low-temperature in grapevine. *PLoS One* 8:e82652. 10.1371/journal.pone.0082652 24376561 PMC3869719

[B171] Roossinck M. J. (2011). The good viruses: viral mutualistic symbioses. *Nat. Rev. Microbiol.* 9 99–108. 10.1038/nrmicro2491 21200397

[B172] Roossinck M. J. (2014). Metagenomics of plant and fungal viruses reveals an abundance of persistent lifestyles. *Front. Microbiol.* 5:767. 25628611 10.3389/fmicb.2014.00767 PMC4290624

[B173] Roossinck M. J. (2015). Plants, viruses and the environment: ecology and mutualism. *Virology* 479–480, 271–277. 10.1016/j.virol.2015.03.041 25858141

[B174] Roossinck M. J. Bazan E. R. (2017). Symbiosis: viruses as intimate partners. *Annu. Rev. Virol.* 4 123–139. 10.1146/annurev-virology-110615-042323 28787582

[B175] Roossinck M. J. Garcia-Arenal F. (2015). Ecosystem simplification, biodiversity loss and plant virus emergence. *Curr. Opin. Virol.* 10 56–62. 10.1016/j.coviro.2015.01.005 25638504 PMC7102708

[B176] Roossinck M. J. Martin D. P. Roumagnac P. (2015). Plant virus metagenomics: advances in virus discovery. *Phytopathology* 105 716–727. 10.1094/PHYTO-12-14-0356-RVW 26056847

[B177] Sahu P. P. Rai N. K. Puranik S. Roy A. Khan M. Prasad M. (2012). Dynamics of defense-related components in two contrasting genotypes of tomato upon infection with tomato leaf curl new delhi virus. *Mol. Biotechnol.* 52 140–150. 10.1007/s12033-011-9481-8 22161255

[B178] Sanfacon H. (2013). Investigating the role of viral integral membrane proteins in promoting the assembly of nepovirus and comovirus replication factories. *Front. Plant Sci.* 3:313. 10.3389/fpls.2012.00313 23439982 PMC3557413

[B179] Sanfacon H. (2015). Plant translation factors and virus resistance. *Viruses* 7 3392–3419. 10.3390/v7072778 26114476 PMC4517107

[B180] Sanfacon H. (2017). Grand challenge in plant virology: understanding the impact of plant viruses in model plants, in agricultural crops, and in complex ecosystems. *Front. Microbiol.* 8:860. 10.3389/fmicb.2017.00860 28596756 PMC5442230

[B181] Santovito E. Mascia T. Siddiqui S. A. Minutillo S. A. Valkonen J. P. Gallitelli D. (2014). Infection cycle of artichoke italian latent virus in tobacco plants: meristem invasion and recovery from disease symptoms. *PLoS One* 9:e99446. 10.1371/journal.pone.0099446 24911029 PMC4050035

[B182] Scholthof H. B. (2006). The *Tombusvirus*-encoded P19: from irrelevance to elegance. *Nat. Rev. Microbiol.* 4 405–411. 16518419 10.1038/nrmicro1395

[B183] Senjam P. Senapati B. K. Chattopadhyay A. Dutta S. (2018). Genetic control of yellow vein mosaic virus disease tolerance in *Abelmoschus esculentus* (L.) Moench. *J. Genet.* 97 25–33. 29666322

[B184] Shan H. Pasin F. Tzanetakis I. E. Simon-Mateo C. Garcia J. A. Rodamilans B. (2017). Truncation of a P1 leader proteinase facilitates *Potyvirus* replication in a non-permissive host. *Mol. Plant Pathol.* 19 1504–1510. 10.1111/mpp.12640 29115017 PMC6638051

[B185] Shan H. Pasin F. Valli A. (2015). The *Potyviridae* P1a leader protease contributes to host range specificity. *Virology* 476 264–270. 10.1016/j.virol.2014.12.013 25562450

[B186] Shapiro L. R. Salvaudon L. Mauck K. E. (2013). Disease interactions in a shared host plant: effects of pre-existing viral infection on cucurbit plant defense responses and resistance to bacterial wilt disease. *PLoS One* 8:e77393. 10.1371/journal.pone.0077393 24155951 PMC3796458

[B187] Shen Q. Hu T. Bao M. (2016). Tobacco RING E3 Ligase NtRFP1 Mediates ubiquitination and proteasomal degradation of a Geminivirus-encoded betaC1. *Mol. Plant* 9 911–925. 10.1016/j.molp.2016.03.008 27018391

[B188] Shen W. Dallas M. B. Goshe M. B. Hanley-Bowdoin L. (2014). SnRK1 Phosphorylation of AL2 delays *Cabbage leaf curl virus* infection in *Arabidopsis*. *J. Virol.* 18 10598–10612. 10.1128/JVI.00761-14 24990996 PMC4178870

[B189] Shi B. Lin L. Wang S. (2015). Identification and regulation of host genes related to *Rice stripe virus* symptom production. *New Phytol.* 209 1106–1119. 10.1111/nph.13699 26487490

[B190] Shimura H. Pantaleo V. Ishihara T. (2011). A viral satellite RNA induces yellow symptoms on tobacco by targeting a gene involved in chlorophyll biosynthesis using the RNA silencing machinery. *PLoS Pathog.* 7:e1002021. 10.1371/journal.ppat.1002021 21573143 PMC3088725

[B191] Shivaprasad P. V. Chen H. M. Patel K. Bond D. M. Santos B. A. Baulcombe D. C. (2012). A microRNA superfamily regulates nucleotide binding site-leucine-rich repeats and other mRNAs. *Plant Cell* 24 859–874. 10.1105/tpc.111.095380 22408077 PMC3336131

[B192] Shukla A. Pagan I. Garcia-Arenal F. (2018). Effective tolerance based on resource reallocation is a virus-specific defence in *Arabidopsis thaliana*. *Mol. Plant Pathol.* 19 1454–1465. 10.1111/mpp.12629 29027740 PMC6638070

[B193] Siddiqui S. A. Sarmiento C. Kiisma M. (2008). Effects of viral silencing suppressors on *Tobacco ringspot virus* infection in two *Nicotiana* species. *J. Gen. Virol.* 89 1502–1508. 10.1099/vir.0.83621-0 18474567

[B194] Simon A. E. Roossinck M. J. Havelda Z. (2004). Plant virus satellite and defective interfering RNAs: new paradigms for a new century. *Annu. Rev. Phytopathol.* 42 415–437. 15283672 10.1146/annurev.phyto.42.040803.140402

[B195] Singh R. P. Burnett P. A. Albarran M. Rajaram S. (1993). Bdv1: a gene for tolerance to *Barley yellow dwarf virus* in bread wheats. *Crop Sci.* 33 231–234.

[B196] Smith N. A. Eamens A. L. Wang M. B. (2011). Viral small interfering RNAs target host genes to mediate disease symptoms in plants. *PLoS Pathog.* 7:e1002022. 10.1371/journal.ppat.1002022 21573142 PMC3088724

[B197] Stare T. Ramsak Z. Blejec A. (2015). Bimodal dynamics of primary metabolism-related responses in tolerant potato-*Potato virus Y* interaction. *BMC Genomics* 16:716. 10.1186/s12864-015-1925-2 26386579 PMC4575446

[B198] Stare T. Stare K. Weckwerth W. Wienkoop S. Gruden K. (2017). Comparison between proteome and transcriptome response in potato (*Solanum tuberosum* L.) leaves following *Potato virus Y* (PVY) infection. *Proteomes* 5:E14. 10.3390/proteomes5030014 28684682 PMC5620531

[B199] Stork J. Panaviene Z. Nagy P. D. (2005). Inhibition of in vitro RNA binding and replicase activity by phosphorylation of the p33 replication protein of *Cucumber necrosis* *Tombusvirus*. *Virology* 343 79–92. 16154612 10.1016/j.virol.2005.08.005

[B200] Su J. Yang L. Zhu Q. (2018). Active photosynthetic inhibition mediated by MPK3/MPK6 is critical to effector-triggered immunity. *PLoS Biol.* 16:e2004122. 10.1371/journal.pbio.2004122 29723186 PMC5953503

[B201] Syller J. Grupa A. (2016). Antagonistic within-host interactions between plant viruses: molecular basis and impact on viral and host fitness. *Mol. Plant Pathol.* 17 769–782. 10.1111/mpp.12322 26416204 PMC6638324

[B202] Szittya G. Silhavy D. Molnar A. (2003). Low temperature inhibits RNA silencing-mediated defence by the control of siRNA generation. *EMBO J.* 22 633–640. 12554663 10.1093/emboj/cdg74 PMC140757

[B203] Tian D. Traw M. B. Chen J. Q. Kreitman M. Bergelson J. (2003). Fitness costs of R-gene-mediated resistance in *Arabidopsis thaliana*. *Nature* 423 74–77. 12721627 10.1038/nature01588

[B204] Trinks D. Rajeswaran R. Shivaprasad P. V. (2005). Suppression of RNA silencing by a Geminivirus nuclear protein, AC2, correlates with transactivation of host genes. *J. Virol.* 79 2517–2527. 15681452 10.1128/JVI.79.4.2517-2527.2005 PMC546592

[B205] Truniger V. Aranda M. A. (2009). Recessive resistance to plant viruses. *Adv. Virus Res.* 75 119–159. 20109665 10.1016/S0065-3527(09)07504-6

[B206] Ustun S. Hafren A. Hofius D. (2017). Autophagy as a mediator of life and death in plants. *Curr. Opin. Plant Biol.* 40 122–130. 10.1016/j.pbi.2017.08.011 28946008

[B207] van Molken T. de Caluwe H. Hordijk C. A. (2012). Virus infection decreases the attractiveness of white clover plants for a non-vectoring herbivore. *Oecologia* 170 433–444. 10.1007/s00442-012-2322-z 22526939 PMC3439618

[B208] van Munster M. Yvon M. Vile D. Dader B. Fereres A. Blanc S. (2017). Water deficit enhances the transmission of plant viruses by insect vectors. *PLoS One* 12:e0174398. 10.1371/journal.pone.0174398 28467423 PMC5414972

[B209] Varallyay E. Havelda Z. (2013). Unrelated viral suppressors of RNA silencing mediate the control of ARGONAUTE1 level. *Mol. Plant Pathol.* 14 567–575. 10.1111/mpp.12029 23578299 PMC6638692

[B210] Varallyay E. Olah E. Havelda Z. (2014). Independent parallel functions of p19 plant viral suppressor of RNA silencing required for effective suppressor activity. *Nucleic Acids Res.* 42 599–608. 10.1093/nar/gkt846 24062160 PMC3874164

[B211] Verchot J. (2016a). How does the stressed out ER find relief during virus infection? *Curr. Opin. Virol.* 17 74–79. 10.1016/j.coviro.2016.01.018 26871502

[B212] Verchot J. (2016b). Plant virus infection and the ubiquitin proteasome machinery: arms race along the endoplasmic reticulum. *Viruses* 8:E314. 27869775 10.3390/v8110314 PMC5127028

[B213] Vidavsky F. Czosnek H. (1998). tomato breeding lines resistant and tolerant to *Tomato yellow leaf curl virus* issued from *Lycopersicon hirsutum*. *Phytopathology* 88 910–914. 10.1094/PHYTO.1998.88.9.910 18944868

[B214] Vogler H. Kwon M. O. Dang V. (2008). *Tobacco mosaic virus* movement protein enhances the spread of RNA silencing. *PLoS Pathog.* 4:e1000038. 10.1371/journal.ppat.1000038 18389061 PMC2270343

[B215] Wang A. (2015). Dissecting the molecular network of virus-plant interactions: the complex roles of host factors. *Annu. Rev. Phytopathol.* 53 45–66. 10.1146/annurev-phyto-080614-120001 25938276

[B216] Wang J. Tang Y. Yang Y. (2016a). Cotton leaf curl Multan virus-derived viral small RNAs can target cotton genes to promote viral infection. *Front. Plant Sci.* 7:1162. 10.3389/fpls.2016.01162 27540385 PMC4972823

[B217] Wang J. Wang X. R. Zhou Q. (2016b). iTRAQ protein profile analysis provides integrated insight into mechanisms of tolerance to TMV in tobacco (*Nicotiana tabacum*). *J. Proteomics* 132 21–30. 10.1016/j.jprot.2015.11.009 26608101

[B218] Wang K. D. Empleo R. Nguyen T. T. Moffett P. Sacco M. A. (2015). Elicitation of hypersensitive responses in *Nicotiana glutinosa* by the suppressor of RNA silencing protein P0 from *Poleroviruses*. *Mol. Plant Pathol.* 16 435–448. 10.1111/mpp.12201 25187258 PMC6638411

[B219] Wang X. Goregaoker S. P. Culver J. N. (2009). Interaction of the *Tobacco mosaic virus* replicase protein with a Nac domain transcription factor is associated with the suppression of systemic host defenses. *J. Virol.* 83 9720–9730. 10.1128/JVI.00941-09 19625399 PMC2748025

[B220] Wang Y. Bao Z. Zhu Y. Hua J. (2009). Analysis of temperature modulation of plant defense against biotrophic microbes. *Mol. Plant Microbe Interact.* 22 498–506. 10.1094/MPMI-22-5-0498 19348568

[B221] Westwood J. H. Lewsey M. G. Murphy A. M. (2014). Interference with jasmonic acid-regulated gene expression is a general property of viral suppressors of RNA silencing but only partly explains virus-induced changes in plant-aphid interactions. *J. Gen. Virol.* 95 733–739. 10.1099/vir.0.060624-0 24362960 PMC3929171

[B222] Westwood J. H. McCann L. Naish M. (2013). A viral RNA silencing suppressor interferes with abscisic acid-mediated signalling and induces drought tolerance in *Arabidopsis thaliana*. *Mol Plant Pathol.* 14 158–170. 10.1111/j.1364-3703.2012.00840.x 23083401 PMC6638696

[B223] Whitham S. McCormick S. Baker B. (1996). The N gene of tobacco confers resistance to *Tobacco mosaic virus* in transgenic tomato. *Proc. Natl. Acad. Sci. U.S.A.* 93 8776–8781. 8710948 10.1073/pnas.93.16.8776 PMC38750

[B224] Wieczorek P. Obrepalska-Steplowska A. (2015). Suppress to survive-implication of plant viruses in PTGS. *Plant Mol. Biol. Rep.* 33 335–346. 25999662 10.1007/s11105-014-0755-8 PMC4432016

[B225] Wu D. Qi T. Li W. X. (2017). Viral effector protein manipulates host hormone signaling to attract insect vectors. *Cell Res.* 27 402–415. 10.1038/cr.2017.2 28059067 PMC5339842

[B226] Wu H. W. Lin S. S. Chen K. C. Yeh S. D. Chua N. H. (2010). Discriminating mutations of HC-Pro of *Zucchini yellow mosaic virus* with differential effects on small RNA pathways involved in viral pathogenicity and symptom development. *Mol. Plant Microbe Interact.* 23 17–28. 10.1094/MPMI-23-1-0017 19958135

[B227] Xu D. Zhou G. (2017). Characteristics of siRNAs derived from Southern rice black-streaked dwarf virus in infected rice and their potential role in host gene regulation. *Virol. J.* 14:27. 10.1186/s12985-017-0699-3 28183327 PMC5301327

[B228] Xu J. Liu D. Zhang Y. (2016). Improved pathogenicity of a beet black scorch virus variant by low temperature and Co-infection with its satellite RNA. *Front. Microbiol.* 7:1771. 10.3389/fmicb.2016.01771 27867378 PMC5095503

[B229] Xu P. Chen F. Mannas J. P. Feldman T. Sumner L. W. Roossinck M. J. (2008). Virus infection improves drought tolerance. *New Phytol.* 180 911–921. 10.1111/j.1469-8137.2008.02627.x 18823313

[B230] Yang J. Y. Iwasaki M. Machida C. Machida Y. Zhou X. Chua N. H. (2008). betaC1, the pathogenicity factor of TYLCCNV, interacts with AS1 to alter leaf development and suppress selective jasmonic acid responses. *Genes Dev.* 22 2564–2577. 10.1101/gad.1682208 18794352 PMC2546693

[B231] Yang S. Hua J. (2004). A haplotype-specific resistance gene regulated by BONZAI1 mediates temperature-dependent growth control in *Arabidopsis*. *Plant Cell* 16 1060–1071. 15031411 10.1105/tpc.020479 PMC412877

[B232] Ye C. Dickman M. B. Whitham S. A. Payton M. Verchot J. (2011). The unfolded protein response is triggered by a plant viral movement protein. *Plant Physiol.* 156 741–755. 10.1104/pp.111.174110 21474436 PMC3177272

[B233] Ye C. M. Chen S. Payton M. Dickman M. B. Verchot J. (2013). TGBp3 triggers the unfolded protein response and SKP1-dependent programmed cell death. *Mol. Plant Pathol.* 14 241–255. 10.1111/mpp.12000 23458484 PMC6638746

[B234] Ye L. Fu X. Ge F. (2010). Elevated CO_2_ alleviates damage from *Potato virus Y* infection in tobacco plants. *Plant Sci.* 179 219–224.

[B235] Yi H. Richards E. J. (2007). A cluster of disease resistance genes in *Arabidopsis* is coordinately regulated by transcriptional activation and RNA silencing. *Plant Cell* 19 2929–2939. 17890374 10.1105/tpc.107.051821 PMC2048694

[B236] Yong Chung H. Lacatus G. Sunter G. (2014). Geminivirus AL2 protein induces expression of, and interacts with, a calmodulin-like gene, an endogenous regulator of gene silencing. *Virology* 460–461, 108–118. 10.1016/j.virol.2014.04.034 25010276

[B237] Yu W. Murfett J. Schoelz J. E. (2003). Differential induction of symptoms in *Arabidopsis* by P6 of *Cauliflower mosaic virus*. *Mol. Plant Microbe Interact.* 16 35–42. 12580280 10.1094/MPMI.2003.16.1.35

[B238] Zamir D. Ekstein-Michelson I. Zakay Y. (1994). Mapping and introgression of a *Tomato yellow leaf curl virus* tolerance gene, TY-1. *Theor. Appl. Genet.* 88 141–146. 10.1007/BF00225889 24185918

[B239] Zhai J. Jeong D. H. De Paoli E. (2011). MicroRNAs as master regulators of the plant NB-LRR defense gene family via the production of phased, trans-acting siRNAs. *Genes Dev.* 25 2540–2553. 10.1101/gad.177527.111 22156213 PMC3243063

[B240] Zhang C. Wu Z. Li Y. Wu J. (2015). Biogenesis, function, and applications of virus-derived small RNAs in plants. *Front. Microbiol.* 6:1237. 10.3389/fmicb.2015.01237 26617580 PMC4637412

[B241] Zhang L. Chen H. Brandizzi F. Verchot J. Wang A. (2015). The UPR branch IRE1-bZIP60 in plants plays an essential role in viral infection and is complementary to the Only UPR pathway in yeast. *PLoS Genet.* 11:e1005164. 10.1371/journal.pgen.1005164 25875739 PMC4398384

[B242] Zhang G. Chen M. Li L. (2009). Overexpression of the soybean GmERF3 gene, an AP2/ERF type transcription factor for increased tolerances to salt, drought, and diseases in transgenic tobacco. *J. Exp. Bot.* 60 3781–3796. 10.1093/jxb/erp214 19602544 PMC2736888

[B243] Zhang L. Wang A. (2012). Virus-induced ER stress and the unfolded protein response. *Front. Plant Sci.* 3:293. 10.3389/fpls.2012.00293 23293645 PMC3531707

[B244] Zhang X. Yuan Y. R. Pei Y. (2006). *Cucumber mosaic virus*-encoded 2b suppressor inhibits *Arabidopsis* Argonaute1 cleavage activity to counter plant defense. *Genes Dev.* 20 3255–3268. 17158744 10.1101/gad.1495506 PMC1686603

[B245] Zhang X. Zhang X. Singh J. Li D. Qu F. (2012). Temperature-dependent survival of *Turnip crinkle virus*-infected *Arabidopsis* plants relies on an RNA silencing-based defense that requires dcl2, AGO2, and HEN1. *J. Virol.* 86 6847–6854. 10.1128/JVI.00497-12 22496240 PMC3393596

[B246] Zhang X. P. Liu D. S. Yan T. (2017). *Cucumber mosaic virus* coat protein modulates the accumulation of 2b protein and antiviral silencing that causes symptom recovery in planta. *PLoS Pathog.* 13:e1006522. 10.1371/journal.ppat.1006522 28727810 PMC5538744

[B247] Zhao J. Zhang X. Hong Y. Liu Y. (2016). Chloroplast in plant-virus interaction. *Front. Microbiol.* 7:1565 10.3389/fmicb.2016.01565 PMC5047884 27757106

[B248] Zhong X. Wang Z. Q. Xiao R. (2017). Mimic phosphorylation of a betaC1 protein encoded by TYLCCNB impairs its functions as a viral suppressor of RNA silencing and a symptom determinant. *J. Virol.* 91:e00300-17. 10.1128/JVI.00300-17 28539450 PMC5533934

[B249] Zhu Y. Qian W. Hua J. (2010). Temperature modulates plant defense responses through NB-LRR proteins. *PLoS Pathog.* 6:e1000844. 10.1371/journal.ppat.1000844 20368979 PMC2848567

[B250] Zorzatto C. Machado J. P. Lopes K. V. (2015). NIK1-mediated translation suppression functions as a plant antiviral immunity mechanism. *Nature* 520 679–682. 10.1038/nature14171 25707794 PMC4779052

[B251] Zvereva A. S. Golyaev V. Turco S. (2016). Viral protein suppresses oxidative burst and salicylic acid-dependent autophagy and facilitates bacterial growth on virus-infected plants. *New Phytol.* 211 1020–1034. 10.1111/nph.13967 27120694

[B252] Zvereva A. S. Pooggin M. M. (2012). Silencing and innate immunity in plant defense against viral and non-viral pathogens. *Viruses* 4 2578–2597. 10.3390/v4112578 23202495 PMC3509663

